# Making multi-twisted luminophores produce persistent room-temperature phosphorescence†

**DOI:** 10.1039/d2sc05741g

**Published:** 2022-12-19

**Authors:** Shen Shen, Glib V. Baryshnikov, Qishan Xie, Bin Wu, Meng Lv, Hao Sun, Zhongyu Li, Hans Ågren, Jinquan Chen, Liangliang Zhu

**Affiliations:** a State Key Laboratory of Molecular Engineering of Polymers, Department of Macromolecular Science, Fudan University Shanghai 200438 China zhuliangliang@fudan.edu.cn; b Laboratory of Organic Electronics, Department of Science and Technology, Linköping University Norrköping 60174 Sweden; c State Key Laboratory of Precision Spectroscopy, East China Normal University Shanghai 200241 P. R. China; d Department of Physics and Astronomy, Uppsala University Box 516 Uppsala SE-751 20 Sweden

## Abstract

Multi-twisted molecules, especially those with more than four branched rotation axes, have served as superior prototypes in diverse fields like molecular machines, optical materials, sensors, and so forth. However, due to excessive non-radiative relaxation of these molecules, it remains challenging to address their persistent room-temperature phosphorescence (pRTP), which limits their further development. Herein, we develop a host–guest energy-transfer relay strategy to improve the phosphorescence lifetime of multi-twisted luminophores by over thousand-fold to realize pRTP, which can be witnessed by the naked eye after removing the excitation light source. Moreover, we employ photoexcitation-induced molecular rearrangement to further prolong the phosphorescence lifetime, which, to the best of our knowledge, is the first example of photoactivation in ordered host–guest systems. Our systems show superior humidity and oxygen resistance, enabling long-term (at least over 9–12 months) stability of the pRTP properties. By achieving pRTP of multi-twisted luminophores, this work can advance the understanding of molecular photophysical mechanisms and guide the study of more molecular systems that are difficult to achieve pRTP.

## Abstract 2

Engineering pRTP of multi-twisted arenes through compatible host–guest (H/G) singlet–singlet and triplet–triplet energy transfers. Moreover, the photoactivation in prolonging pRTP lifetime is the first reported photoactivation in ordered H/G systems.

## Graphical abstract

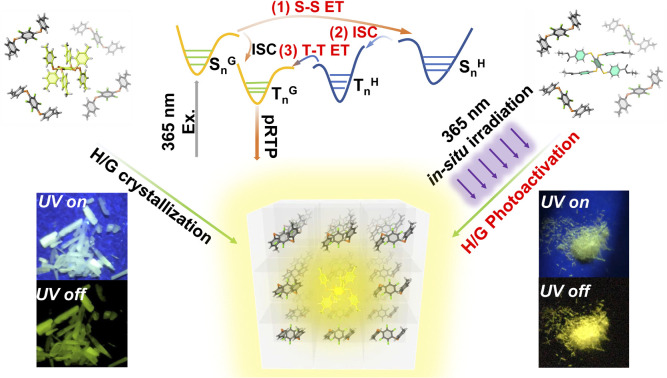

## Introduction

Multi-twisted molecules refer to a large class of structures with multiple twisted conformations, such as molecular rotors,^1–3^ molecules with quasi-axial/equatorial multi-conformations,^4,5^ asterisk molecules,^6,7^*etc.* Such a conformational feature endows them with broad application prospects in molecular machines and motors,^8,9^ optical materials,^10,11^ sensors,^12,13^ and so forth. For photophysical aspects, multi-twisted molecules are extensively investigated in aggregation-induced emission luminogens (AIEgens), thermally activated delayed fluorescence (TADF) materials, and stimuli-responsive materials, but their persistent room-temperature phosphorescence (pRTP) has been less attended. pRTP refers to a photoluminescence phenomenon that can be observed with the naked eye after removing the excitation light source.^14,15^ Usually, the pRTP lifetime needs to be longer than 10 ms to be witnessed.^16^ Currently, well-developed organic pRTP materials generally include luminophores with rigid or planar conformations, such as fluorene derivatives,^17,18^ carbazole derivatives,^19–21^ dibenzothiophenes,^22–24^ heteroatom-containing planar aromatic compounds,^25–27^*etc.* In contrast, multi-twisted molecules are prone to exert excessive non-radiative relaxation, unstable triplet excitons, and lack of rigidity, which lead to huge difficulties in realizing pRTP and therefore hamper a variety of applications.^28,29^

Doping guest emitters into hosts is conducive to the construction of pRTP systems,^30–32^ whereas molecular twisting requires more considerations of structural, heavy atom, and energy level compatibility. The improvement of phosphorescence quantum efficiency (*Φ*_p_) requires a fast intersystem crossing rate (*K*_ISC_) and large spin-orbit coupling (SOC), which refer to the conditions of the heavy atom effect, the EL-Sayed rule, and the energy gap. However, these conditions can also accelerate the phosphorescence radiative decay rate (*K*_phos._) and reduce the phosphorescence lifetime.^33–35^ In this way, moderate heavy atom effects and eligible energy gaps are likely to strike a balance between the RTP lifetime and quantum efficiency,^35,36^ which is desired to develop a rational and systematic strategy for the pRTP of twisted luminophores.

**Fig. 1:**
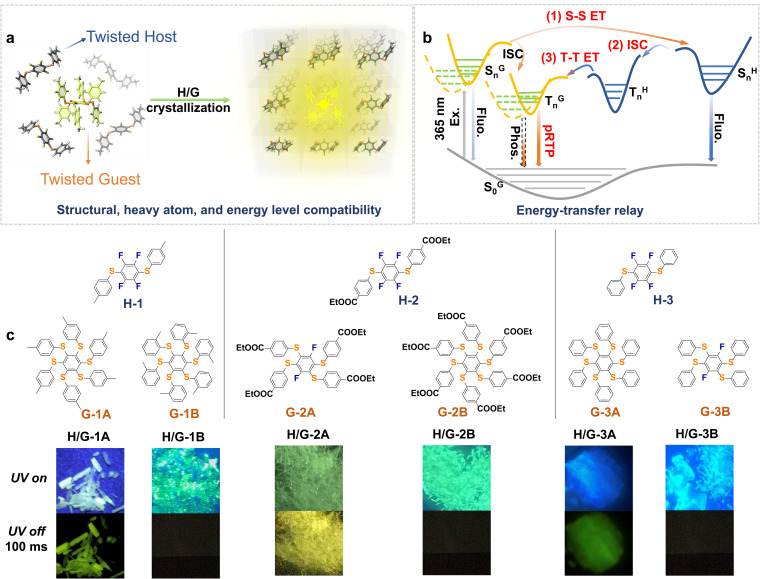
(a) Schematic representation of the general H/G strategy of multi-twisted luminophores upon crystallization. (b) The main idea to obtain pRTP in the H/G crystal systems with energy-transfer relay (Ex., excitation; Fluo., fluorescence; Phos., phosphorescence; S^G^_*n*_, excited singlet states of the guest; T^G^_*n*_, excited triplet states of the guest; S^H^_*n*_, excited singlet states of the host; T^H^_*n*_, excited triplet states of the host, *n* ≥ 1). (c) Molecular structures of the hosts and guests and photographs of a series of typically constructed H/G crystals under (UV on) and after (UV off: 100 ms) 365 nm excitation, featuring the necessity of energy levels and structural compatibility.

Persulfurated arenes are a typical class of multiple twisted molecules that generally adopt asterisk conformations with a high degree of distortion (normally 4–6 branched rotation axes to exert excessive non-radiative relaxation).^6,37^ This structural characteristic makes it more difficult for the molecules to achieve pRTP.^38,39^ Moreover, the abundant sulfur atoms in tetra/hexasubstituted persulfurated arenes can enhance the *Φ*_p_,^6,40–42^ while simultaneously accelerating the S_*n*_–T_*n*_ and T_*n*_–S_0_ electronic transitions to produce shortened phosphorescence lifetimes in the order of microseconds.^33,43^ As demonstrated in Fig. 1a, we propose to realize their pRTP through constructing host–guest (H/G) crystal systems of twisted hosts and twisted guests and to create an energy-transfer (ET) relay effect for enabling successive singlet–singlet (S–S) and triplet–triplet (T–T) ETs, based on available energy levels and rigidification conditions (Fig. 1b). We designed and synthesized a series of twisted disubstituted arenes as hosts. Then, we selected severely twisted tetra- or hexa-substituted arenes as guests.^44,45^ A small amount of guests (about 0.3–6.0% mol ratio of guest) is doped into the hosts by co-crystallization, which is beneficial to confine the luminophore with abundant intermolecular F⋯H and interlayer π–π interactions to ensure the ET relay. The energy gap between the singlet state of the guest and the singlet state of the host (Δ*E*_S^G^–S^H^_) plays a crucial role in defining the energy matching. Accordingly, a series of highly stable H/G pRTP systems (H/G-1A, H/G-2A, and H/G-3A) were eventually developed (Fig. 1c), while the remaining pairs cannot exhibit pRTP due to the mismatch of energy levels and lack of structural compatibility. Furthermore, inspired by the photoactivation for prolonging the phosphorescence lifetime that has been reported on single component or amorphous polymer-doped systems,^19,46^ we applied a photoexcitation-induced molecular rearrangement strategy into a H/G-2A system, which further improved the phosphorescence lifetime by more than 10 fold, realizing the first example of photoactivation in ordered host–guest systems.

## Results

### Construction of the H/G crystal systems

The hosts and guests in this work are designed based on the principle of structural similarity to access energy level compatibility. Small amounts of highly purified guest emitters were crystalized into highly purified hosts to form a series of H/G systems. The loading molar ratio of H/G compounds is *ca.* 1 : 0.15% (see the actual H/G doping ratio study *vide infra*). All of the crystals exhibited blue to yellowish emissions under a 365 nm UV lamp (Fig. 1c). After the UV lamp was turned off, remarkable pRTP with a green or yellow color could be observed in H/G-1A, H/G-2A and H/G-3A (Fig. 1c). In addition to the three best results, weak pRTP can also be found in some cross-doped H/G crystals. For example, as shown in Table S1,† when G-3A is doped in H-1, the obtained crystal exhibits weak pRTP with a lifetime in the range of 1–10 ms, which still possesses a longer phosphorescence lifetime than the sole host or sole guest. These results indicate the universality of this strategy in prolonging the RTP lifetime. In contrast, the remaining pairs cannot exhibit pRTP due to the mismatch of energy levels and lack of structural compatibility.

Powder X-ray diffraction (PXRD) shows that the characteristic diffraction pattern of H/G crystals is not only derived from the mixed signals of the host and guest, but also accompanied by the emergence of new peaks (Fig. S1†), indicating the generation of a new crystal form. However, such trace guest content does not reach the detection range of single crystal characterization, leaving the host as the only detected component in H/G single crystals. As shown in Fig. S2,† the peripheral substituents of all the hosts are distributed nearly perpendicularly on both sides of the plane of the inner benzene core, resulting in a two-dimensional symmetric framework.

On this basis, intermolecular π–π and F⋯H interactions are extended along the *c*-axis into an ordered three-dimensional porous stacking,^47,48^ suggesting that the accommodation space provided by such a twisted conformation can facilitate the insertion of guests. Moreover, the intermolecular F⋯H and interlayer π–π interactions between the host and the guest are conducive to confining the molecular motion of the guest.

### H/G doping ratio

**Fig. 2:**
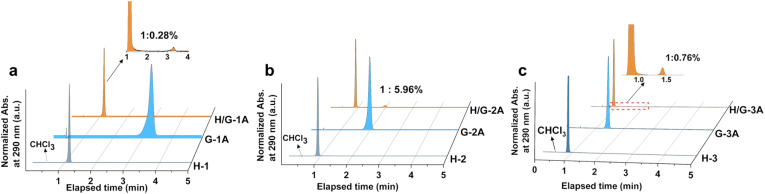
HPLC spectra recorded at the onset absorption of 290 nm for (a) H-1, G-1A, H/G-1A, (b) H-2, G-2A, and H/G-2A; (c) H-3, G-3A, and H/G-3A.

The purity of hosts and guests as well as the H/G doping ratio are crucial factors to determine photophysical properties;^32^ therefore, high-performance liquid chromatography (HPLC) was employed for analysis. After optimizing the polarity in the eluent, an appropriate ratio (90/10) of acetonitrile/water was used for the separation and characterization of hosts and guests through monitoring the UV-vis absorption at 290 nm in HPLC, which is benefited from strong absorption of both hosts and guests (Fig. S3†). The host and guest compounds were strictly screened by HPLC to ensure their own purity (Fig. 2a–c and S4†), which benefits from the three times purification by silica gel column chromatography before crystallization. The optimal doping amount is selected from adjusting the doping ratio. There exists an optimal doping weight ratio of 1 : 0.3% for constructing the pRTP system, which causes the longest phosphorescent lifetime upon changing the dopant ratio of guests (Fig. S5†). The HPLC results show that the actual H/G doping mol ratio of H/G-1A, H/G-2A and H/G-3A is 1 : 0.28%, 1 : 5.96%, and 1 : 0.76%, respectively, revealing a vital feature with a small doping amount of guests in constructing the pRTP systems. In addition, a discernible resonance is generated in NMR of H/G-2A, relative to H/G-1A and H/G-3A, because the ethyl ester groups can enlarge the push–pull electron effect between H-2 and G-2A. As shown in Fig. S6,† the integral areas demonstrate a doping molar ratio of 1 : 5% in H/G-2A, which is close to the result shown in HPLC and provides an additional confirmation of the H/G doping ratio.^49^ The confirmed H/G ratios are different from the loading mol ratio (*ca.* 1 : 0.15%) for crystallization, suggesting the existence of a spontaneous crystallization process. The crystallized H/G crystals were collected instantly to avoid guest or host deposition and perturbation.

### Photophysical properties

**Fig. 3:**
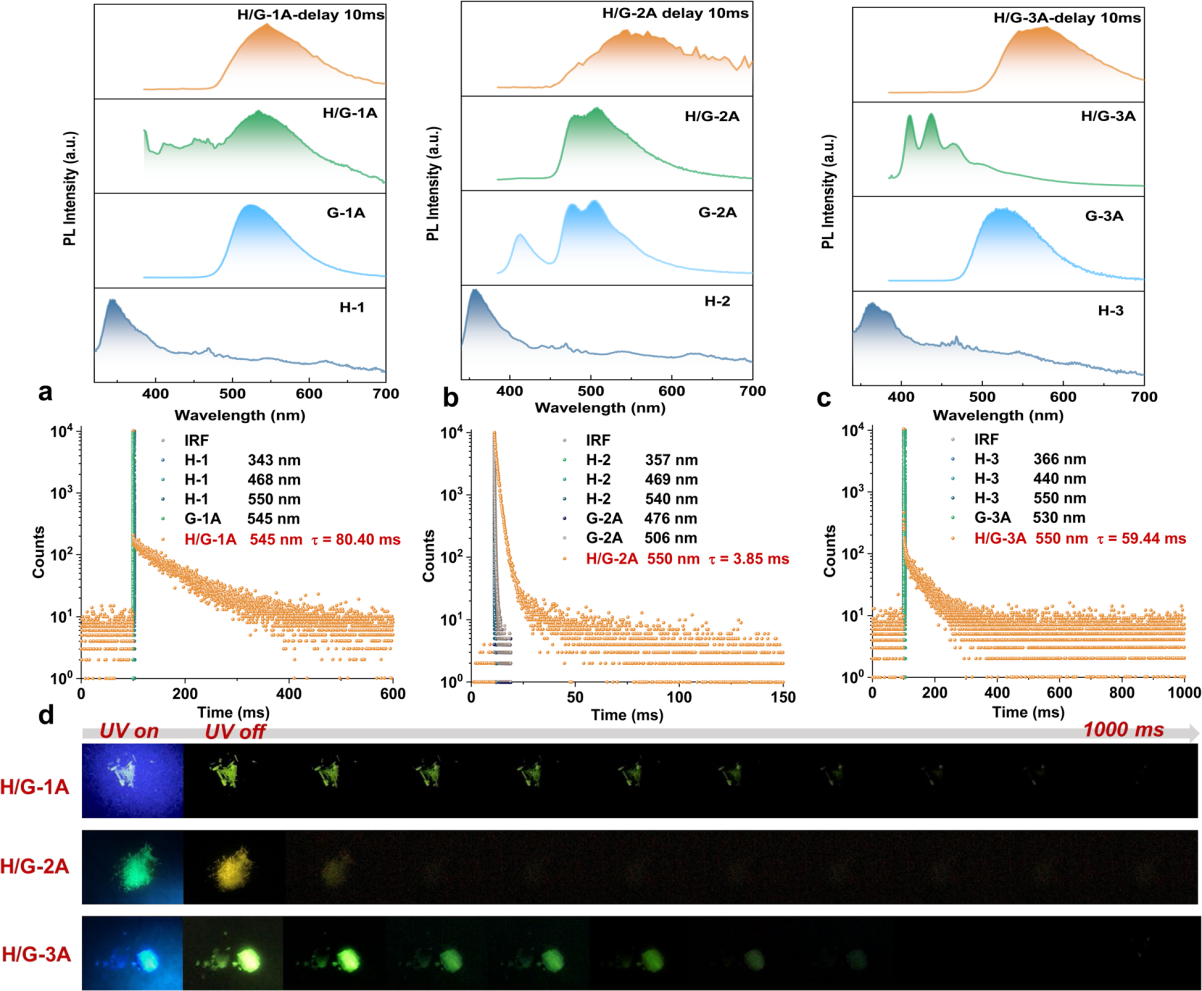
(a) The steady-state PL spectra of H-1 (*λ*_ex_ = 293 nm), G-1A (*λ*_ex_ = 365 nm) and H/G-1A (*λ*_ex_ = 365 nm) and corresponding PL decay spectra. (b) The steady-state PL spectra of H-2 (*λ*_ex_ = 320 nm), G-2A (*λ*_ex_ = 365 nm) and H/G-2A (*λ*_ex_ = 365 nm) and corresponding PL decay spectra. (c) The steady-state PL spectra of H-3 (*λ*_ex_ = 293 nm), G-3A (*λ*_ex_ = 365 nm) and H/G-3A (*λ*_ex_ = 365 nm) and corresponding PL decay spectra. (d) The photographs of crystal samples of H/G-1A, 2A, and 3A before and after turning off the 365 nm UV irradiation.

The photophysical properties of the three typical pRTP systems were meticulously studied both spectroscopically and theoretically. According to the calculated S_0_–S_*n*_ absorption (Table S2†) and solid UV-vis absorption spectra (Fig. S7†), none of the hosts can be excited at 365 nm. Upon appropriate excitation (293 nm for H-1 and H-3, and 320 nm for H-2), all the hosts exhibit short-wavelength emission with phosphorescence lifetimes below 15 μs and quantum yields (*Φ*_PL_) below 1% (Fig. 3a–c and S8†). The hexa-substituted guests G-1A and G-3A show a *Φ*_PL_ of 85% and 42% with a green phosphorescence lifetime of 3.23 μs and 14.05 μs under 365 nm excitation, respectively (Fig. 3a and c and S8†). The tetra-substituted G-2A exhibits turquoise phosphorescence with a relatively long lifetime of 253.26 μs and a lower *Φ*_PL_ of 6% under 365 nm excitation (Fig. 3b and S8†). Interestingly, their phosphorescence lifetime was largely boosted in the H/G systems. Delayed emission spectra (delay time = 10 ms and *λ*_ex_ = 365 nm) show that the pRTP bands all center at *ca.* 550 nm with a lifetime of 80.40 ms, 3.85 ms and 59.44 ms for H/G-1A, H/G-2A, and H/G-3A, respectively (Fig. 3a–c). As shown in Fig. S9,† the pRTP decay of H/G-1A did not change upon 350 nm and 365 nm excitation. However, the pRTP lifetime of H/G-3A at 365 nm excitation is longer than that of 350 nm excitation. Although the absorption band at 365 nm in H/G-1A and H/G-3A is not remarkable due to the small amounts of doping, we chose 365 nm excitation for these systems related to the optimal excitation of the guests. Furthermore, the wavelengths of the delayed emission bands are very close to those of the guests, indicating that the pRTP is generated from triplet excitons of the guests. Once excited at 293 nm, the H/G systems (H/Gs) only emit weak host emission without a prolongated RTP lifetime. These facts demonstrate that selective excitation is crucial in these host–guest pRTP systems. The excitation cessation process of H/Gs under ambient conditions was recorded by using a camera (Fig. 3d), which clearly exhibits the pRTP witnessed by the naked eye after removing the excitation light source.

Considering the fact that persulfurated arenes (non-salt form) did not exhibit pRTP after being doped into polyvinyl alcohol (a widely used confining matrix to prolong PL lifetimes),^28^ it can be concluded that the enhanced phosphorescence lifetime in H/Gs cannot be entirely attributed to the restriction of molecular motion. As verified by cryogenic (77 K) spectroscopic characterization (Fig. S10†), crystalline H-3 and G-3A produced phosphorescence emission with a lifetime of 5.00 ms and 12.69 ms, respectively. This is far from the pRTP lifetime of H/G-3A at room temperature (59.44 ms), indicating that there are possible ETs between the host and guest to facilitate the generation of long-lived triplet excitons. As the conformation of persulfurated arenes is crucial for their phosphorescent properties,^37^ the similar conformations of H-1 to H-3 and G-1A to G-3A (Fig. S11†) indicate that their photoluminescence properties are close. As verified in Fig. 3, H/G-1A and H/G-3A have similar pRTP lifetimes (80.40 ms *vs.* 59.44 ms) and pRTP emissions (545 nm *vs.* 550 nm). In combination with the T_1_ state of the chromophores localized on the inner C6S6 core (Tables S3 and S4†) and the minor simulated conformational differences (Table S5†), as well as the fact that G-1A already has been analyzed from a computational point of view,^50^ we selected two representative H/G-2A and H/G-3A systems for theoretical calculations. All the structures for calculation are extracted from single crystal data.

**Fig. 4:**
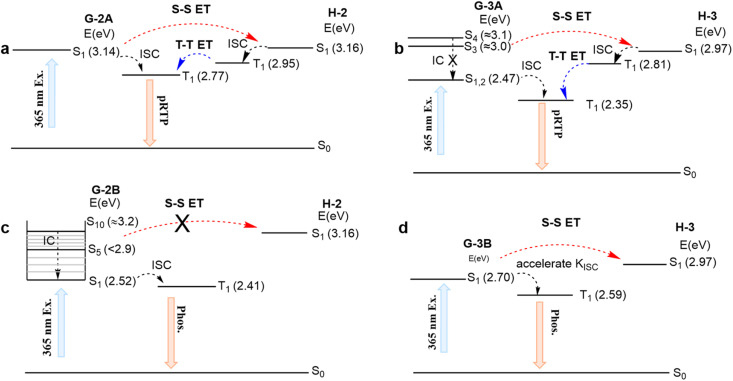
Calculated PL mechanism of (a) H/G-2A, (b) H/G-3A, (c) H/G-2B and (d) H/G-3B. Abbreviations ISC, IC, S–S ET and T–T ET correspond to the intersystem-crossing, internal conversion, singlet–singlet energy transfer and triplet–triplet energy transfer, respectively.

The energies of the relaxed S_1_ states for both H-2 and G-2A components are in close resonance (*i.e.*, Δ*E*_S^G^–S^H^_ is very small, Fig. 4a). This means that upon excitation of H/G-2A with 365 nm light, which thus is in close resonance with vertical S_0_–S_1_ absorption of G-2A, an efficient ET can be achieved from the S_1_ state of G-2A to the S_1_ state of H-2, followed by relaxation into the T_1_ state of H-2 through efficient ISC. Then, an efficient and accelerated T–T ET from H-2 to G-2A is observed because of the energy level comparability with a Δ*E*_T^H^–T^G^_ of 0.18 eV, leading to the subsequent delayed phosphorescence (*K*_phos._ is estimated to be 7 × 10^2^ s^−1^ for H/G-2A conf 1, Table S6† and Fig. 4a). The UV-vis absorption and emission spectra of H-2 and G-2A in acetonitrile solution are shown in Fig. S12.† The emission of guests reveals fluorescence–phosphorescence multiple bands in solution, verified by the insensitivity of shorter wavelength emission to oxygen and sensitivity of longer wavelength emission to oxygen in Fig. S13.† Here, the fluorescence bands at shorter wavelengths are in resonance with tail absorption of the hosts, indicating that the guest-to-host S–S ET can occur *via* Förster resonance energy transfer (FRET). The T–T ET process can be achieved *via* a short-range Dexter mechanism (the distance between the host and guest <1 nm), which can be verified in the crystalline spectra. For examples, the H-2 emission (see the <400 nm band in Fig. 3b, also verified as a phosphorescence by the PL lifetime study in Fig. S8†) resonates with the absorption of G-2A (Fig. S7†). This fact indicates a large possibility in the systems to cause efficient host-to-guest T–T ET (Table S7†). These processes form a closed loop of the ET relay to finally generate pRTP. Similarly, such an ET relay is valid in H/G-3A. In terms of the photoexcitation at 365 nm that can reach higher singlet states (*e.g.*, the S_4_ state, *ca.* 3.10 eV), an S–S ET process can still occur to reach the S_1_ state of H-3 (*ca.* 2.97 eV) because of the small Δ*E*_S^G^–S^H^_ (0.13 eV) (Fig. 4b) and FRET (Fig. S12†). Then, a significant part of the energy endures ISC to the T_1_ state. Subsequently, a slow T–T ET with a Δ*E*_T^H^–T^G^_ of 0.46 eV from T_1_ of H-3 to T_1_ of G-3A occurs, thus limiting the rate of phosphorescence radiative decay for G-3A (*K*_phos._ is estimated to be 4 × 10^2^ s^−1^ for H/G-3A conf 3, Table S6†), *i.e.*, prolonging the phosphorescence lifetime up to *ca.* 60 ms.

After photoexcitation of H/G-2B, G-2B can reach S_*n*_ (*n* > 5) states. The vibrational relaxation into an S_*n*_ minimum state can support the intramolecular cascade S_*n*_–S_1_ internal conversion instead of S–S ET (Fig. 4c). Upon excitation of 365 nm for H/G-3B, only the S_1_ state with 2.70 eV of G-3B can be reached to endure accelerated ISC rather than S–S ET. Thus, the low-lying G-2B and G-3B have difficulty in spontaneously ET to the corresponding high-lying hosts. Thus, H/G-2B and H/G-3B systems emit only guest emission without pRTP upon excitation at 365 nm. According to the above analyses, the mentioned mechanism of H/G-3A can also be applicable to H/G-1A. However, G-1B with multiple conformations (Fig. S11e†) was prone to undergo vibrational relaxation rather than S–S ET. In addition, compared with G-1B with the methyl group on the inside, the outside methyl group of G-1A can well prevent strong intermolecular interactions, resulting in slower T–T ET. These results explain that some of the cross-doped H/G crystal systems with eligible energy levels can also prolong the RTP lifetime. Overall, three prerequisites need to be satisfied in the established general strategy: (1) selective excitation of the guest; (2) a small Δ*E*_S^G^–S^H^_; (3) H/G doping with molecular structures bearing suitable heavy atoms to balance ISC and SOC. In addition, the pRTP could be well sustained when these crystals were placed under ambient conditions even for 9–12 months (Fig. S14†), indicating that these H/G systems possess high stability against moisture and oxygen. From this perspective, they can be superior to traditional pRTP systems for practical applications.^51,52^

### Photoactivation

**Fig. 5:**
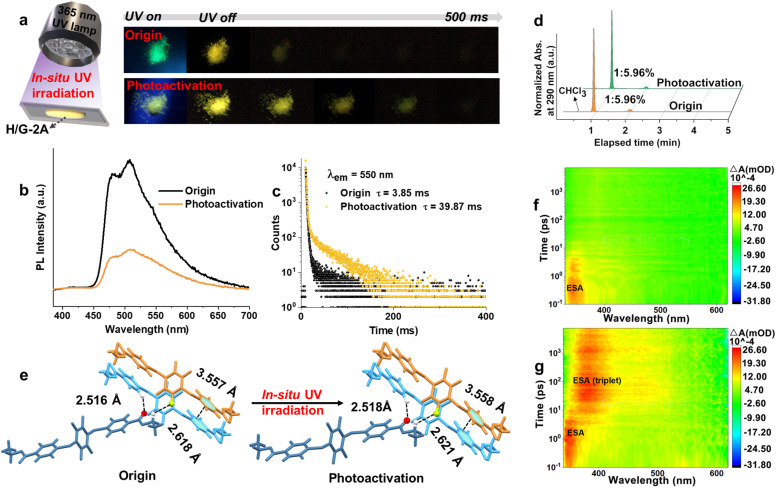
(a) *In situ* UV (365 nm) irradiation of H/G-2A and the photographs of H/G-2A before and after photoactivation for 3 h. (b) PL spectra and (c) corresponding decay curves of H/G-2A before and after photoactivation. (d) HPLC spectra of H/G-2A before and after photoactivation for 3 h monitored at 290 nm. (e) The changes of intermolecular crystal interaction in H/G-2A crystals before and after photoactivation. 2D color plots of the TA spectra of the H/G-2A crystal film (f) before and (g) after photoactivation for 3 h under 320 nm excitation.

In view of the photoexcitation-induced molecular realignment properties of tetra-arylthiolbenzenes that we reported earlier,^44^*in situ* photoirradiation of the H/G-2A system containing tetra-arylthiolbenzenes was performed. Upon irradiation at 365 nm under ambient conditions for 0.5 h (3.85 mW cm^−1^), a further prolonged phosphorescence lifetime from 3.85 ms to 14.79 ms was observed (Fig. S15†), *i.e.*, a photoactivation process occurred. After 3 h of photoactivation, a more remarkable pRTP was observed (Fig. 5a), and the lifetime was prolonged by 10.35 fold from 3.85 ms to 39.87 ms (Fig. 5b and c). Simultaneously, the declined *Φ*_PL_ from 41% to 4% indicated that the *K*_phos._ and SOC have been altered. The pRTP properties of H/G-2A after photoactivation are also observed to be very stable, since no further variation of the lifetime could be observed after placing under ambient conditions for 12 months (Fig. S16†) or heating treatment (Fig. S17†). The invariant HPLC (Fig. 5d) and NMR (Fig. S18,† as compared to Fig. S6†) spectra before and after UV irradiation, which exhibit no additional signals corresponding to new substances, validated that there was no photochemical process occurring during the *in situ* UV irradiation. In addition, an oxygen-consuming process can be ruled out because the same photoactivation phenomenon could also be seen with *in situ* UV irradiation in a vacuum. Therefore, a physics-based process, photoexcitation-induced molecular realignment, may take place in this photoactivation process.

To further verify this realignment process, X-ray crystallography was employed to visualize the sample before and after irradiation. As the trace amount of G-2A cannot be detected by single crystal characterization in H/G-2A, the photoexcitation-induced molecular realignment process was characterized from H-2. As shown in Fig. 5e, upon irradiation, the intermolecular distance of C=O⋯H increases from 2.516 Å to 2.518 Å, and the intermolecular distance of F⋯H increases from 2.618 Å to 2.621 Å, as well as the interlayer π–π distance increases from 3.557 Å to 3.558 Å. In view of the ordered periodic system of single crystals, these parameter changes are reasonable to induce an amplification effect with detectable changes in photophysical properties.^44^ It has been found that the T–T ET of pristine H/G-2A is efficient and fast, which contributes to relatively short-lived triplet excitons (T–T energy gap is 0.18 eV, as shown in Fig. 4a). This fact, combined with the tighter intermolecular distances, indicates that there is a more developed contact interface between the H-2 and G-2A phases before irradiation (Fig. S19†). The increased distance indicates that the packing is looser along with the realignment of G-2A upon UV irradiation, which may perturb the well-developed interfaces (Fig. S19†). In addition, after UV irradiation, the PXRD characteristic peak intensity declined at 12.54° and increased at 16.46° (Fig. S20†). The former peak coincides with the characteristic peak of G-2A, and the latter characteristic peak is present in both H-2 and G-2A, so the tradeoff in peak intensity further verifies that the insertion disturbs the developed contact interfaces. Herein, the loose packing and disturbance in the well-developed interfaces contribute to ‘traps’ like stochastic defects where the excitation can sustain for a long time,^53^ resulting in slowing T–T ET and thus further prolonging the pRTP lifetime (Fig. S19†). Heating or cooling treatment cannot cause the same stable pRTP (Fig. S21†).

After UV irradiation, the solid-state UV absorption band of H/G-2A is broadened and red-shifted (Fig. S22†). An excitation–emission mapping shows that the excitation center after UV irradiation is also significantly broadened and that a red-shifted tail band at 620 nm emerges (Fig. S23†), indicating an enhanced generation of triplet excitons. To gain in-depth insight into the unique optical properties, femtosecond transient absorption (TA) and theoretical calculations were performed.^54^ For the H/G-2A crystal film, only one excited-state absorption (ESA) band, at ∼340 nm decaying within 5 ps, could be found (Fig. 5f and S24a†). After UV irradiation, the ESA signals at ∼340 nm decayed within 10 ps, accompanied by an emerged red-shifted ESA band at 380–500 nm, which decayed gradually over the next 8 ns (Fig. 5g and S24b†). The decay time of these two ESA signals (under vacuum measurement conditions) as well as their trade-off trend show a slower T–T ET. According to the red-shifted phosphorescence emission (513 nm, Table S6†) and the smaller π–π overlap of H/G-2A conf 2 (Fig. S25†), it is reasonable to take the photoactivation state as H/G-2A conf 2, because of the red-shifted emission and looser packing after photoactivation.

### Writing/patterning information encoding

**Fig. 6:**
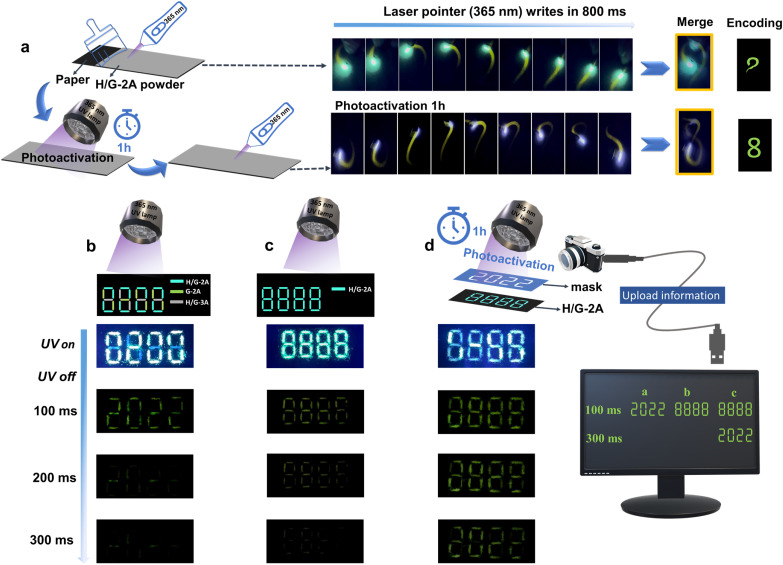
(a) The fabrication of UV (365 nm) laser-rewritable paper and the writing process of an encoded pattern. (b) The pRTP photographs of H/G-2A, G-2A and H/G-3A powder crystals (distributed in different regions of an “8888” pattern) at 100 ms, 200 ms and 300 ms after the stoppage of 365 nm excitation. (c) The pRTP photographs of an “8888” pattern with H/G-2A powder crystals only at 100 ms, 200 ms and 300 ms after the stoppage of 365 nm excitation. (d) The 100 ms, 200 ms and 300 ms pRTP photographs of H/G-2A powder crystals after 1 h of photoactivation in the selective region (mask applied) of an “8888” pattern after the stoppage of 365 nm excitation.

The pRTP stability, specific emission lifetime and intensity, and the pRTP photoactivation prolonging characteristics controlled by UV irradiation give an intriguing possibility to use the constructed H/G systems for writing/patterning information encoding.^55^ To realize this proposition, we first ground the crystals of H/G-2A into powders and stuck them uniformly on a non-fluorescent paper (Fig. 6a). Using a camera to record the writing traces of a UV (365 nm) laser pen, pRTP traces can be found under a certain writing speed, which can then be merged to show a commas character within 800 ms (Fig. 6a and Video S1†). After photoactivation for 1 h, longer traces can be found within 800 ms, which were merged to show a complete 8-character (Fig. 6a and Video S2†). This writing application indicates a good processability and applicability of the H/G crystals for patterning information encoding. Then, the “8888” pattern was formed using powder crystals of G-2A, H/G-2A and H/G-3A at different positions. As shown in Fig. 6b, the pattern shows “8888” upon the irradiation of a 365 nm UV lamp. Once removing the light irradiation for 100 ms, clear “2022” words can be captured by the camera, because of the pRTP properties of H/G-2A and H/G-3A (Video S3†). In the second case, the word “8888” was fabricated solely by the H/G-2A powder crystals and was shown to exhibit bright green-yellow emission under UV excitation of 365 nm (Fig. 6c). After cessation of the UV light, the pattern of “8888” can be observed within 100–200 ms and recorded by the camera (Video S4†). In the third case, a mask with hollow “2022” was placed on the top of the “8888” pattern, and UV irradiation of 365 nm was applied for 1 h for photoactivation (Fig. 6d). After removing the mask and turning off the UV light, the materials generated distinct green pRTP with the information of “8888” within 100 ms. By extending the capture time, the “2022” words are discovered at 300 ms (Video S5†), demonstrating a complex encoding effect. The patterning information of the materials in the article can be cycled for at least hundreds of times. As shown in Fig. S26,† the emission intensities decrease with UV irradiation slightly, whereas the pRTP lifetimes are maintained well. These results indicate that the capture by the camera or the naked eye would be hardly affected. These results highlight the application potential of these H/G systems, and thus they provide more material choices for information encoding and storage, which enrich time-resolved applications.

## Conclusions

A series of multi-twisted luminophore-based H/G pRTP systems were successfully constructed, where an energy-transfer relay based on structural, heavy atom, and energy level compatibility (a small Δ*E*_S^G^–S^H^_, 0–0.1 eV) played a key role. This strategy is conducive to the phosphorescence lifetime promotion of the multi-twisted luminophores by over thousand-fold to realize pRTP. Moreover, we employed *in situ* UV irradiation to further increase the pRTP lifetime of some systems by means of photoexcitation-induced molecular realignment and trapping mechanisms, which is, to the best of our knowledge, the first discovery of photoactivation of ordered H/G systems. Considering the excellent stability of the pRTP systems under ambient conditions, as well as the specific emission lifetime and intensity, the application in writing/patterning information encoding can be fulfilled and guide more probing in time-resolved photoluminescent events. We believe that the strategy demonstrated herein can be valuable for broadening the future development of molecular photophysics and phosphorescent materials.

## Data availability

The authors confirm that the data supporting the findings are available within the article and its ESI material.† Raw data that support the findings of this study are available from the corresponding author, upon reasonable request.

## Author contributions

L. Z. and S. S. conceived the idea and designed the study. S. S. synthesized, purified and crystallized all compounds and performed general photophysical measurements with the assistance of Q. X. and B. W. G. B. and H. Å. provided theoretical calculations and analyzed the mechanism. M. L. and J. C. performed transient absorption characterization. S. S. analyzed all the single crystal data and drew the schematic diagram with the assistance of B. W. The NMR analysis is supervised and assisted by Z. L. The manuscript was written jointly by L. Z. and S. S and revised with the help from H. S. and H. Å. All authors discussed the results and commented on the manuscript.

## Conflicts of interest

There are no conflicts to declare.

## Supplementary Material

SC-014-D2SC05741G-s001

SC-014-D2SC05741G-s002

SC-014-D2SC05741G-s003

SC-014-D2SC05741G-s004

SC-014-D2SC05741G-s005

SC-014-D2SC05741G-s006

SC-014-D2SC05741G-s007

SC-014-D2SC05741G-s008
