# Supplementary material for: Making multi-twisted luminophores produce persistent room-temperature phosphorescence
Source: Chem Sci. 2022 Dec 19;14(4):970–8. doi: 10.1039/d2sc05741g (PMC9890967; doi:10.1039/d2sc05741g)
Supplement: SC-014-D2SC05741G-s001 [file SC-014-D2SC05741G-s001.pdf]

Electronic Supplementary Material (ESI) for Chemical Science.
This journal is © The Royal Society of Chemistry 2023

# Electronic Supplementary Information

## Making Multi-Twisted Luminophores Produce Persistent Room-Temperature Phosphorescence

Shen Shen,[a] Glib V. Baryshnikov,[b] Qishan Xie,[a] Bin Wu,[a] Meng Lv,[c] Hao Sun,[a] Zhongyu Li,[a] Hans Ågren,[d] Jinquan Chen,[c] Liangliang Zhu*[a]

[a] State Key Laboratory of Molecular Engineering of Polymers, Department of Macromolecular Science, Fudan University, Shanghai 200438, China. Email: zhuliangliang@fudan.edu.cn.

[b] Laboratory of Organic Electronics, Department of Science and Technology, Linköping University, Norrköping, 60174 Sweden.

[c] State Key Laboratory of Precision Spectroscopy, East China Normal University, Shanghai, 200241, P. R. China.

[d] Department of Physics and Astronomy, Uppsala University, Uppsala, Box 516, SE-751 20, Sweden.

Table of Contents

1. Experimental Procedures.......... S2
2. Supporting Figures .......... S5
3. Supporting Tables .......... S29
4. Reference .......... S35

## Experimental Procedures

### 1.1 Materials and Solvents

*p*-Toluenethiol, *o*-Toluenethiol, sodium thiophenolate and perfluorobenzene, used in the experiment were purchased from Energy Chemical, 4-Mercaptobenzoic acid is purchased from Aladdin, all of which were used without further purification. Unless other mentioned, all the solvents were available from Greagent and used directly.

### 1.2 Measurements

Photoluminescence (PL) spectra and decay spectra as well as phosphorescence-gated spectra were obtained from an Edinburgh FLS-1000 luminescence spectrometer equipped with a xenon lamp, a microsecond flashlamp as the excitation source and picosecond pulsed diode lasers with several wavelength as the excitation sources. The Instrument Response Function (IRF) was collected using silica solution as the reference standard. The absolute photoluminescent quantum yields were collected on a QM40 from Photo Technology International, Inc. (PTI, USA), using the integrating sphere method. Powder X-ray diffraction (PXRD) data were collected using a Bruker D8 ADVANCE. The solid UV-vis absorption spectra were recorded on a Perkin-Elmer Lambda 750 spectrophotometer equipment with integrating sphere. Femtosecond transient absorption (TA) spectra and kinetics were measured using a TA spectrometer (Helios fire, Ultrafast System). The fundamental pulses were generated from a femtosecond laser system (Astrella, 800ns, 100fs, 7mJ/pulse, and 1 kHZ repetition rate, Coherent, Inc.), arising two split beams. 800 nm fundamental beam, which was split into two beams. One was passed through an optical parametric amplifier (OPerA Solo, Coherent Inc.) to generate the pump beam, and the other generated a white light continuous probe beam in a commercial TA spectrometer (Helios fire, Ultrafast System). The polarization between pump and probe beams is set to be 54.7°. The excitation wavelength was adjusted to be 320 nm. The IRF was determined to be ~120 fs. The $^{1}$H NMR, $^{19}$F NMR and $^{13}$C NMR were recorded on a Bruker 400L spectrometer in chloroform-*d* and DMSO-$d_6$ using tetramethylsilane as internal standard. Mass spectra were acquired on a Matrix Assisted Laser Desorption Ionization-Time of Flight/Time of Flight (MALDI-TOF) Mass Spectrometer (5800). HPLC purification were performed on Agilent 1260 equipped with YMC CombiScreen ODS-AM column CCAMS05-0546WT AM-300-CC (50 x 4.6 mm I.D.). The flow rate was fixed at a rate of 2 mL/min, the injection volume was 20 μL and each sample was run for 10 min. The prepared samples concentration in chloroform ($CHCl_3$) for HPLC analysis were 2 mg $mL^{-1}$. Single crystal X-ray diffraction were measured by a Bruker D8 Venture operating at 173 K, and their structures were resolved and analyzed with the assistance of shelx-2014 software. Optical photoluminescence images were taken under UV (365 nm) light with a Sony camera with burst and video modes.

### 1.3 Computational Details

The structures of H-2, G-2A, G-2B, H-3, G-3A, G-3B were initially optimized at the DFT level by using B3LYP functional, 6-311g(d,p) basis set and Grimme's empirical dispersion correction (GD3) in gas phase approximation. As we have shown previously for G-3A[1] and G-3B[2] derivatives, the cam-B3LYP functional performs better for excited state calculations because of charge-transfer origin of the lowest-lying $S_1$ and $T_1$ states. Thus, for optimizing the $S_1$ and $T_1$ states we have used the GD3-cam-B3LYP/6-311g(d,p) approach by using the spin-restricted time-dependent (TD) DFT method for singlets and spin-unrestricted method for triplets. Following the same methodology as for individual H-2, G-2A, G-2B, H-3, G-3A, G-3B molecules, the dimer complexes of H/G-2A, H/G-2B, H/G-3A and H/G-3B were optimized in the ground singlet ($S_0$), first singlet ($S_1$) and first triplet ($T_1$) states in different space configurations (with different mutual orientation of dimer components). Based on the optimized $S_1$ and $T_1$ state geometries of individual compounds and their dimers we have characterized the fluorescence and phosphorescence parameters by using the cam-B3LYP/TZP[3,4] method implemented in the ADF software (version 2021.107).[5] Scalar relativistic TDDFT[6] calculations within the Tamm-Dancoff approximation (TDA)[6] were performed to determine the energy of lowest $S_1$ and $T_1$ excited states and the spin-orbit coupling (SOC). The ZORA[7,8] operator was then applied to obtain the excitation energies with SOC effects treated as a perturbation (pSOC-TDDFT)[9] based on $S_1$ and $T_1$ geometries for fluorescence and phosphorescence, respectively. SOC matrix elements (SOCMEs) between $S_1$ state and triplet ($T_n$) states lying below $S_1$ state, $\langle S_1|\hat{H}_{SO}|T_n\rangle$, were also computed along pSOC-TDDFT calculations as root mean squares, i.e. as square root of the sum of squares of spin-orbit coupling matrix elements of all triplet state sublevels ($m$=0,±1) of the uncoupled states:

$$\langle S_1|\hat{H}_{SO}|T_n\rangle = \sqrt{\sum_{m=0,\pm1}\langle S_1|\hat{H}_{SO}|T_n^m\rangle^2} \quad (1)$$

The fluorescence rate constants ($k_r$) were estimated according to the following relationship (expressed in atomic units):

$$k_r = \frac{1}{\tau} = \frac{2(\Delta E^2)f}{c^3}, \quad (2)$$

where $\tau$ is radiative life of $S_1$ state, $\Delta E$ and $f$ – the energy and intensity of the corresponding singlet-singlet or singlet-triplet transitions with accounting of SOC perturbations.

The rate constants of intersystem crossing (ISC) between the $S_1$ and $T_j$ states $E(S_1)>E(T_j)$ were estimated using the Plotnikov's empirical approximation:[10]

$$k_{S_1\to T_n} = 10^{10}\langle S_1|\hat{H}_{so}|T_j\rangle^2 F_{0j} \quad (3)$$

where Franck–Condon factors ($F_{0j}$) were approximated using the formula:

$$F_{0j} = \sum_n \prod_v \frac{e^{-y}y^{n_v}}{n_v!} \quad (4)$$

In Equation (5) the Huang–Rhys factor $y$ was assumed to be equal to 0.3 and only one average promotive mode $\omega_v$ = 1400 cm$^{-1}$ was used when considering $n_v = \Delta E(S_1 - T_j)/\omega_v$. Such a single-mode approximation was considered efficient and sufficiently accurate for the organic dyes and fluorophores.[11,12]
Phosphorescence energy and lifetime was additionally estimated by using $T_1$ state geometries. By using Eq. 2 the decay rates for the individual spin-sublevels of the $T_1$ state ($k_m$=1/$\tau^m$) were estimated and then $k_{phos.}=\sum_m k_m$ was calculated.

## 1.4 Synthesis and preparation

Synthesis of Ethyl 4-mercaptobenzoate. Ethyl 4-mercaptobenzoate was synthesized according to reference.[13]

General methods for the synthesis and purification of H-1,[14] H-2. As schematic in Scheme S1, the thiol compound (*p*-Toluenethiol, ethyl 4-mercaptobenzoate) in 2 equivalent was dissolved in dry DMF and added dropwise to 1 equivalent of perfluorobenzene and $K_2CO_3$ in DMF solution and then stirred at room temperature for 12 h. Distilled water was added to obtain the precipitate and washed with ethanol to give white powder. The resulting powder was purified by silica gel column chromatography of petroleum ether (PE) three times and then crystallized from acetonitrile (ACN) to obtain transparent crystals of H-1 and H-2, respectively. Using sodium thiophenolate as thiol compound to synthesize H-3,[14] and the synthetic route is the same as above, without adding $K_2CO_3$.

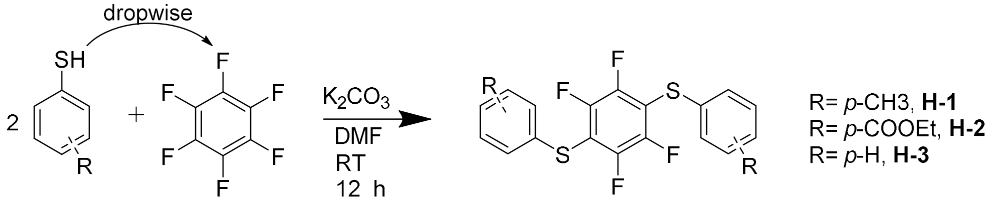


**Scheme S1.** The synthesis route of di-substituted arylthiol benzenes (H-1, H-2, H-3).

1,4-bis(*p*-tolylthio)-2,3,5,6-tetrafluorobenzene (H-1). Column chromatography (PE), transparent weak blue fluorescence crystals. $^1$H NMR (400 MHz, $CDCl_3$) δ = 7.35 (d, *J*=8.2, 4H), 7.14 (d, *J*=7.9, 4H), 2.35 (s, 6H) ppm. $^{19}$F NMR (400 MHz, $CDCl_3$) δ = -132.51 ppm. $^{13}$C NMR (101 MHz, $CDCl_3$) δ = 148.4 – 145.4 (m), 138.6, 132.0, 130.2, 128.9, 21.0 ppm. MS: MALDI-TOF MS, m/z [M]$^+$: calculated for $C_{20}H_{14}F_4S_2$, 394.05; found m/z [M]$^+$, 394.15.

1,4-bis(4-ethylesterphenylthio)-2,3,5,6-tetrafluorobenzene (H-2). Column chromatography (PE/EA 20:1, v/v), transparent weak blue fluorescence crystals. $^1$H NMR (400 MHz, $CDCl_3$) δ = 7.99 (d, J = 8.3 Hz, 4H), 7.36 (d, J = 8.4 Hz, 4H), 4.39 (q, J = 7.1 Hz, 4H), 1.40 (t, J = 7.1 Hz, 6H) ppm. $^{19}$F NMR (400 MHz, $CDCl_3$) δ = -130.63 ppm. $^{13}$C NMR (101 MHz, $CDCl_3$) δ = 165.7, 148.4 – 145.8 (m), 138.6, 130.5, 129.7, 129.0, 61.1, 14.4 ppm. MS: MALDI-TOF MS, m/z[M]$^+$: calculated for $C_{24}H_{18}F_4O_4S_2$, 510.06; found m/z [M+Na]$^+$, 532. 87.

1,4-bis(phenylthio)-2,3,5,6-tetrafluorobenzene (H-3). Column chromatography (PE), transparent weak blue fluorescence crystals. $^1$H NMR (400 MHz, $CDCl_3$) δ = 7.44 – 7.38 (m, 4H), 7.35 – 7.29 (m, 6H) ppm. $^{19}$F NMR (400 MHz, $CDCl_3$) δ = -132.17 ppm. $^{13}$C NMR (101 MHz, $CDCl_3$) δ = 148.0 – 145.8 (m), 132.8, 130.6, 129.1, 127.8 ppm. MS: MALDI-TOF MS, m/z [M]$^+$: calculated for $C_{18}H_{10}F_4S_2$ 366.02; found m/z [M]$^+$, 365.98.

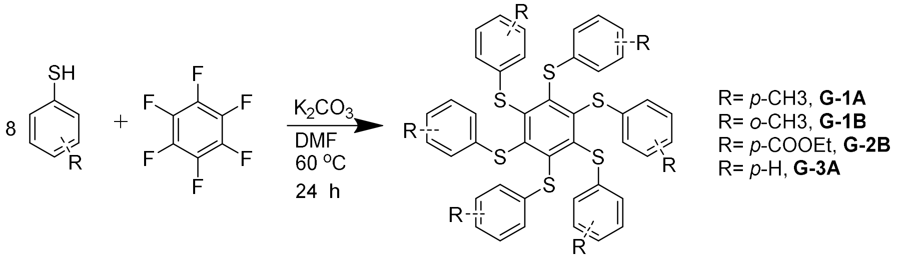


**Scheme S2.** The synthesis route of hexa-substituted arylthiol benzenes (G-1A, G-1B, G-3A).

General methods for the synthesis and purification of G-1A,[15] G-1B, G-2B,[13] G-3A.[1,16] As schematic in Scheme S2, the thiol compound (*p*-Toluenethiol, *o*-Toluenethiol, ethyl 4-mercaptobenzoate), $K_2CO_3$ in 8 equivalent was dissolved in dry DMF and added to 1 equivalent of perfluorobenzene in DMF solution and then stirred at 60 °C for 24 h. Distilled water was added to obtain the precipitate and washed with ethanol to give yellowish powder. The resulting powder was purified by silica gel column chromatography three times and then crystallized from acetonitrile (ACN) to obtain crystals of G-1A, G-1B, G-2B, respectively. Using sodium thiophenolate as thiol compound to synthesize G-3A, and the synthetic route is the same as above, without adding $K_2CO_3$.

Hexa(*p*-tolylthio)-benzene (G-1A). Column chromatography (PE/DCM 10:1, v/v), yellow emission crystals. $^1$H NMR (400 MHz,

$CDCl_3$) δ = 6.95 (d, *J*=8.1, 12H), 6.85 (d, *J*=8.1, 12H), 2.30 (s, 18H) ppm. $^{13}C$ NMR (101 MHz, $CDCl_3$) δ = 148.0, 135.6, 134.3, 129.8, 128.4, 20.8 ppm. MS: MALDI-TOF MS, m/z $[M]^+$: calculated for $C_{48}H_{42}S_6$, 810.16; found m/z $[M+H]^+$, 811.07.

Hexa(*o*-tolylthio)-benzene (G-1B). Column chromatography (PE/EA 10:1, v/v), yellow-green fluorescence crystals. $^1H$ NMR (400 MHz, $CDCl_3$) δ = 7.00 (m, 18H), 6.66 (d, *J*=7.7, 6H), 2.21 (s, 18H) ppm. $^{13}C$ NMR (101 MHz, $CDCl_3$) δ = 148.0, 137.1, 136.2, 130.4, 128.2, 126.2, 125.6, 20.5 ppm. MS: MALDI-TOF MS, m/z $[M]^+$: calculated for $C_{48}H_{42}S_6$, 810.16; found m/z $[M]^+$, 810.23.

Hexa(4-ethylesterphenylthio)-benzene (G-2B). Column chromatography (PE/EA 20:1, v/v), yellow emission crystals. $^1H$ NMR (400 MHz, $CDCl_3$) δ = 7.91 – 7.84 (m, 12H), 7.00 – 6.92 (m, 12H), 4.38 (q, *J*=7.1, 12H), 1.42 (t, *J*=7.1, 18H) ppm. $^{13}C$ NMR (101 MHz, $CDCl_3$) δ = 165.7, 148.0, 142.3, 130.2, 128.4, 126.8, 61.1, 14.4 ppm. MS: MALDI-TOF MS, m/z $[M]^+$: calculated for $C_{60}H_{54}O_{12}S_6$ 1158.19; found m/z $[M+H]^+$, 1159.27.

Hexa(4-phenylthio)-benzene (G-3A). Column chromatography (PE/DCM 10:1, v/v), yellow-green fluorescence crystals. $^1H$ NMR (400 MHz, $CDCl_3$) δ = 7.20 – 7.09 (m, 18H), 7.01 – 6.91 (m, 12H) ppm. $^{13}C$ NMR (101 MHz, $CDCl_3$) δ = 142.7, 135.9, 129.1, 128.1, 126.5 ppm. MS: MALDI-TOF MS, m/z $[M]^+$: calculated for $C_{42}H_{30}S_6$ 726.07; found m/z $[M]^+$, 726.22.

General methods for the synthesis and purification of G-2A, G-3B[2,17]. As schematic in Scheme S3, the thiol compound (ethyl 4-mercaptobenzoate), $K_2CO_3$ in 4 equivalent was dissolved in dry DMF and added to 1 equivalent of perfluorobenzene in DMF solution and then stirred at 60 °C for 24 h. Distilled water was added to obtain the precipitate and washed with ethanol to give white powder. The resulting powder was purified by silica gel column chromatography three times and then crystallized from acetonitrile (ACN) to obtain crystals of G-2A. Using sodium thiophenolate as thiol compound to synthesize G-3B, and the synthetic route is the same as above, without adding $K_2CO_3$.

1,2,4,5-tetra(4-ethylesterphenylthio)-3,6-difluorobenzene (G-2A). Column chromatography (PE/EA 20:1, v/v), blue-greenish fluorescence crystals. $^1H$ NMR (400 MHz, $CDCl_3$) δ = 7.90 (d, J = 8.5 Hz, 8H), 7.17 (d, J = 8.4 Hz, 8H), 4.38 (q, J = 7.1 Hz, 8H), 1.40 (t, J = 7.1 Hz, 12H) ppm. $^{19}F$ NMR (400 MHz, $CDCl_3$) δ = -89.05 ppm. $^{13}C$ NMR (101 MHz, $CDCl_3$) δ = 165.9, 159.6 (dd, $J_1$ = 253.8 Hz, $J_2$ = 4.5 Hz), 140.2, 130.4, 129.1, 128.6 (dd, $J_1$ = 17.1 Hz, $J_2$ = 5.5 Hz), 128.0, 61.3, 14.2 ppm. MS: MALDI-TOF MS, m/z$[M]^+$: calculated for $C_{42}H_{36}F_2O_8S_4$, 834.13; found m/z $[M+Na]^+$, 857.69.

1,2,4,5-tetra(4-phenylthio)-3,6-difluorobenzene (G-3B). Column chromatography (PE/DCM 20:1, v/v), blue fluorescence crystals. $^1H$ NMR (400 MHz, $CDCl_3$) δ = 7.24 – 7.17 (m, 20H) ppm. $^{19}F$ NMR (400 MHz, $CDCl_3$) δ = -90.57 ppm. $^{13}C$ NMR (101 MHz, $CDCl_3$) δ = 159.3 (dd, $J_1$ = 256.6 Hz, $J_2$ = 4.5 Hz), 134.7, 129.5, 129.1, 128.9, 127.0 ppm. MS: MALDI-TOF MS, m/z $[M]^+$: calculated for $C_{30}H_{20}F_2S_4$ 546.04; found m/z $[M]^+$, 546.11.

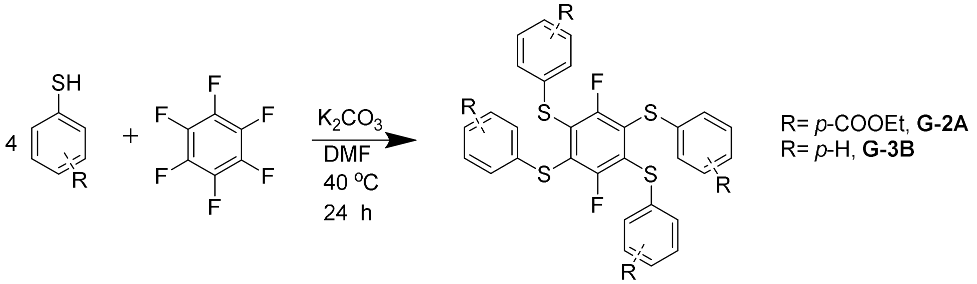


**Scheme S3.** The synthesis route of tetra-substituted arylthiol benzenes (G-2A, G-3B).

## Supporting Figures

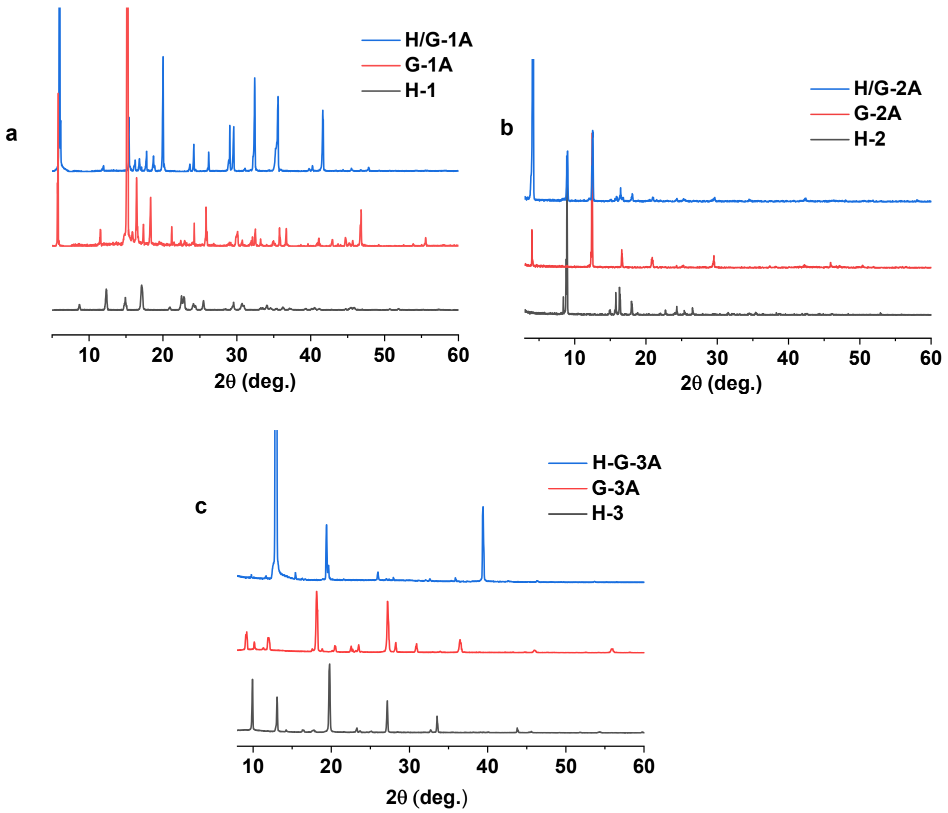


**Figure S1.** (a) PXRD of H-1, G-1A and H/G-1A. (b) PXRD of H-2, G-2A and H/G-2A. (c) PXRD of H-3, G-3A and H/G-3A.

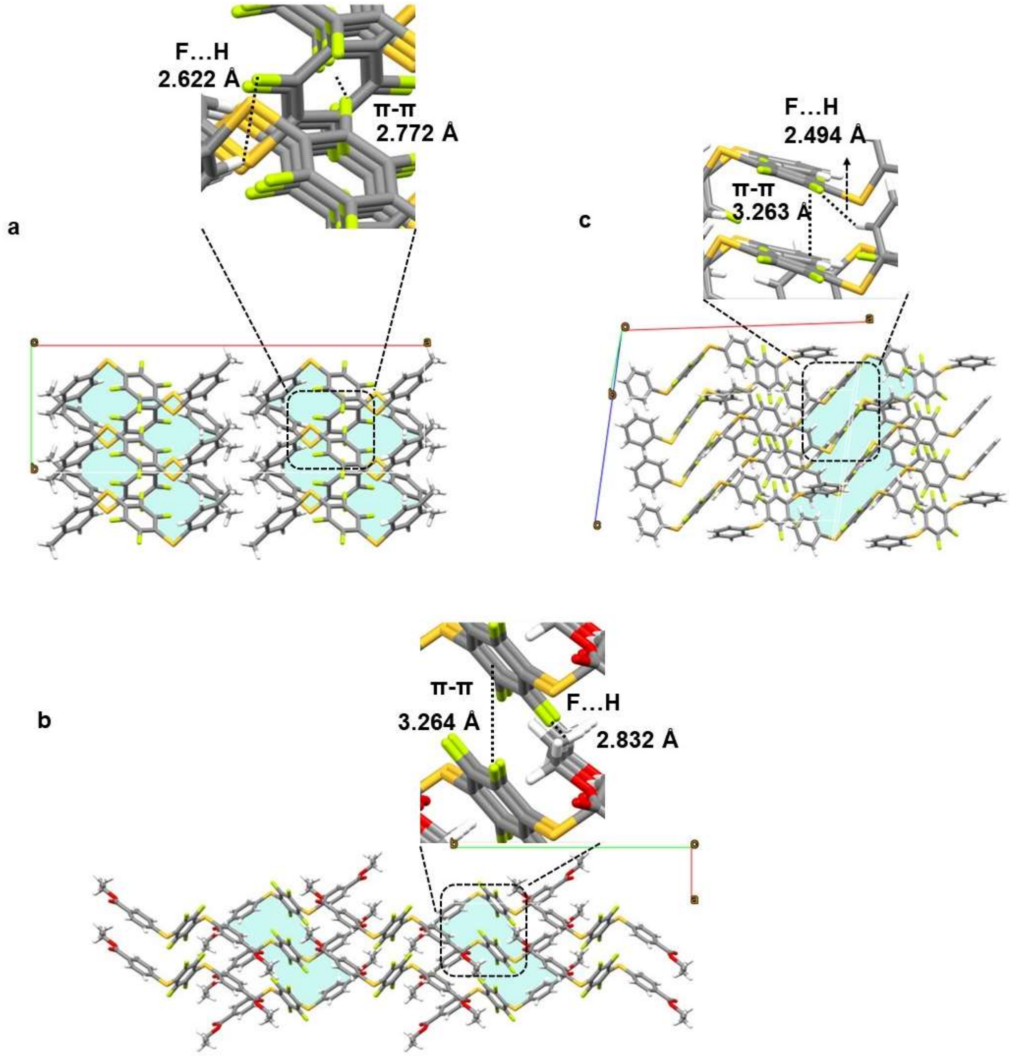


**Figure S2.** (a) Crystal 2x2x2 packing of H-1 along c axis. (b) Crystal 2x2x2 packing of H-2 along c axis. (c) Crystal 1.5x2x2 packing of H-3 along c axis to b axis at 45$^{o}$.

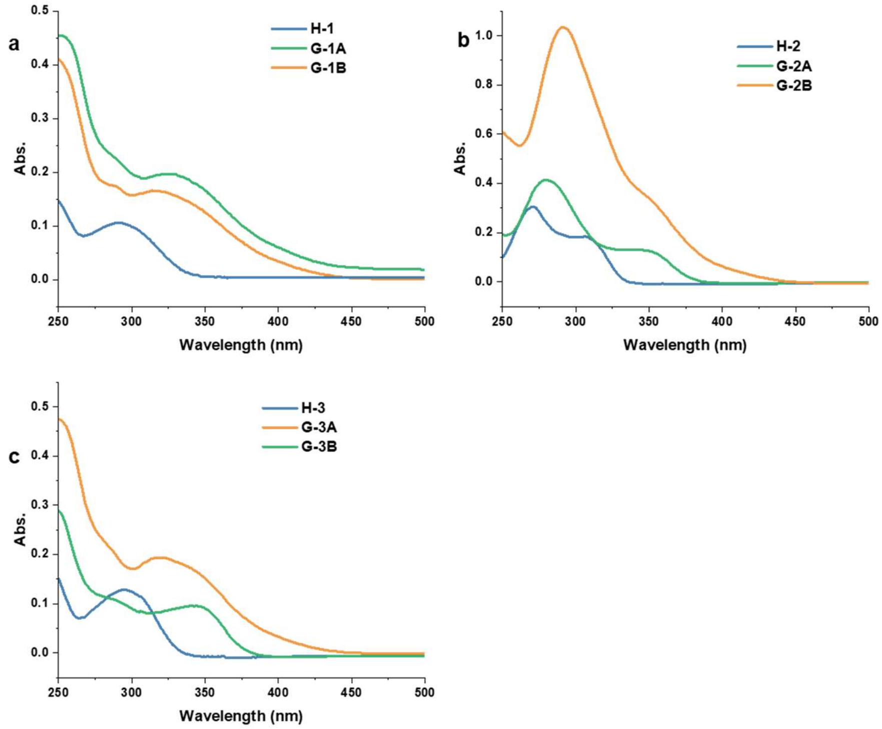


**Figure S3.** UV-vis absorption of H-1, G-1A, G-1B and H/G-1A (a); H-2, G-2A, G-2B and H/G-2A (b); H-3, G-3A, G-3B and H/G-3A (c) in an eluent with $V_{ACN/H2O}$ = 9:1 (20 μM).

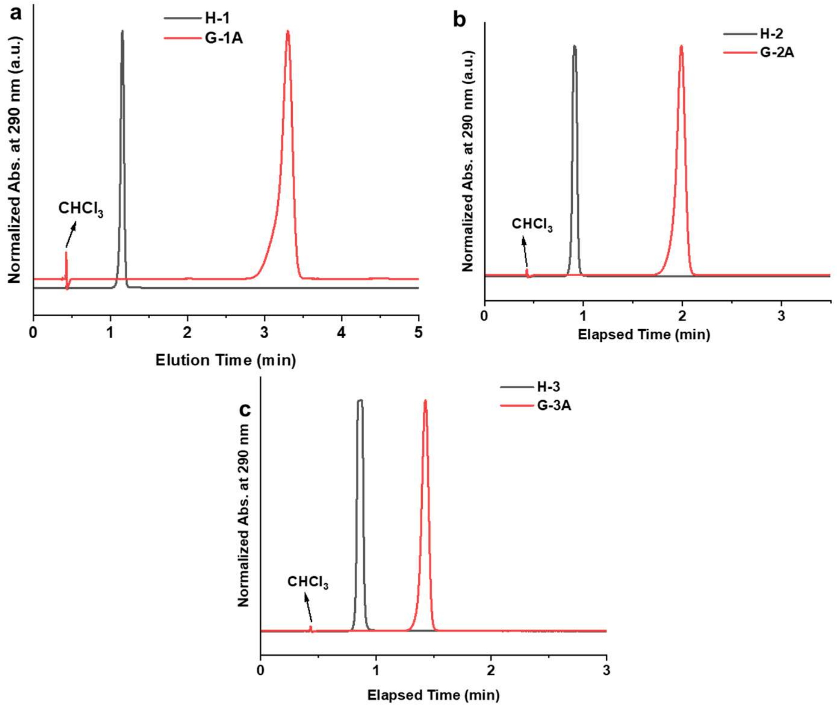


**Figure S4.** HPLC spectra recorded at the onset absorption of 290 nm for (a) H-1, G-1A; (b) H-2, G-2A; (c) H-3, G-3A.

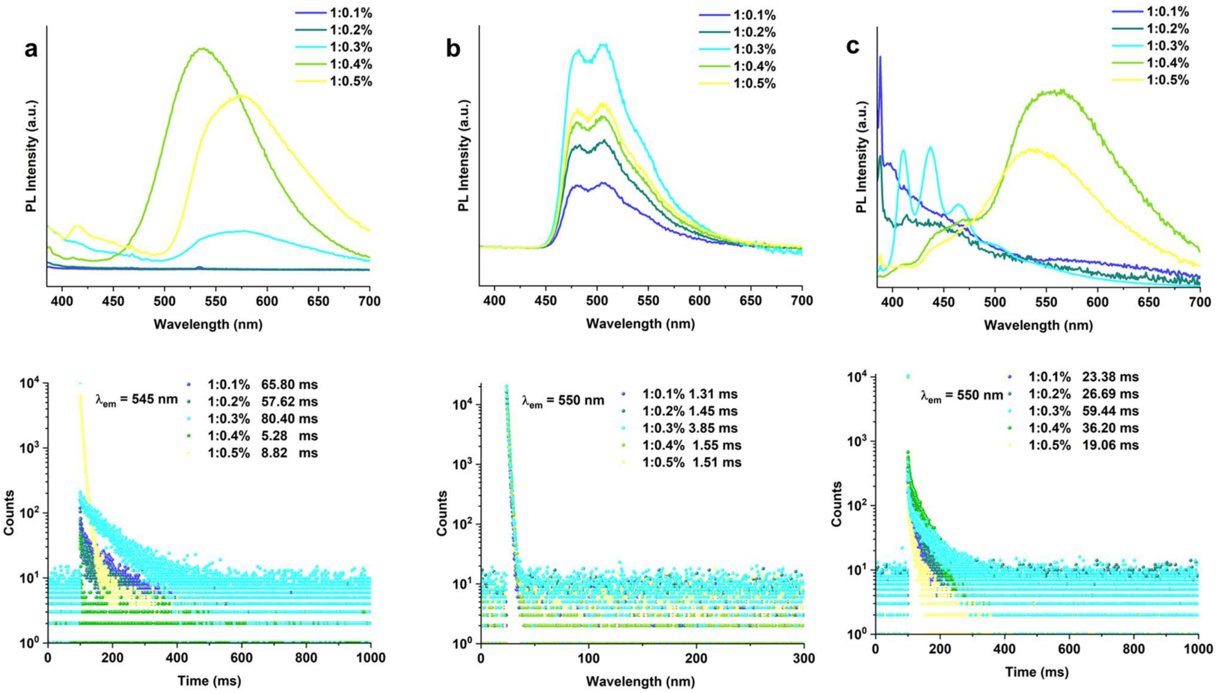


**Figure S5.** The steady-state PL spectra ($\lambda_{ex}$ = 365 nm) of (a) H/G-1A, (b) H/G-2A and (c) H/G-3A and corresponding PL decay spectra with varying host/guest loading weight ratio (1:0.1%, 1:0.2%, 1:0.3%, 1:0.4%, 1:0.5%).

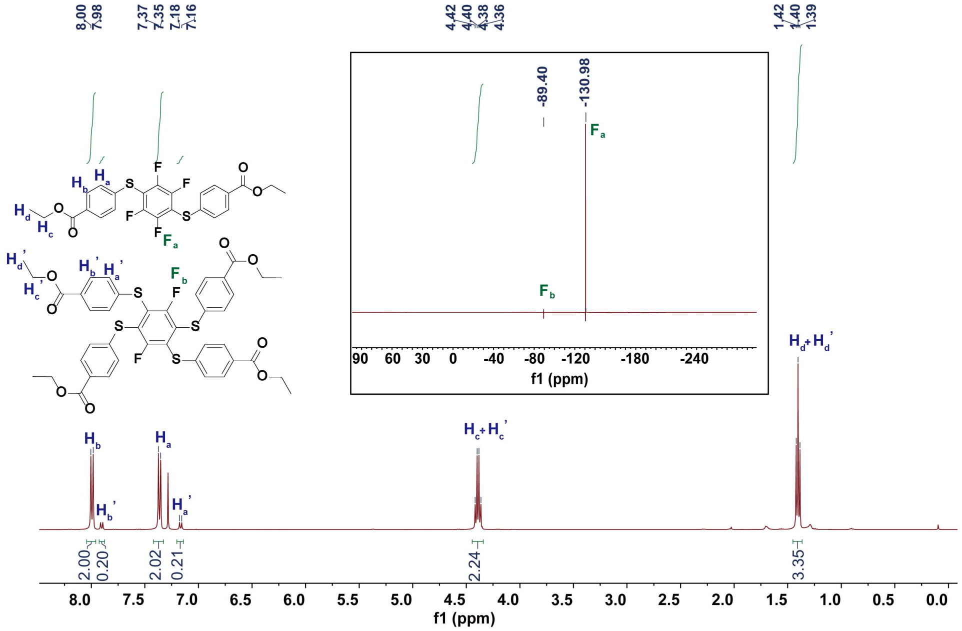


**Figure S6.** $^1$H NMR spectra of H/G-2A and calculated doping ratio based on the integral areas. Insert: $^{19}$F NMR spectra of H/G-2A.

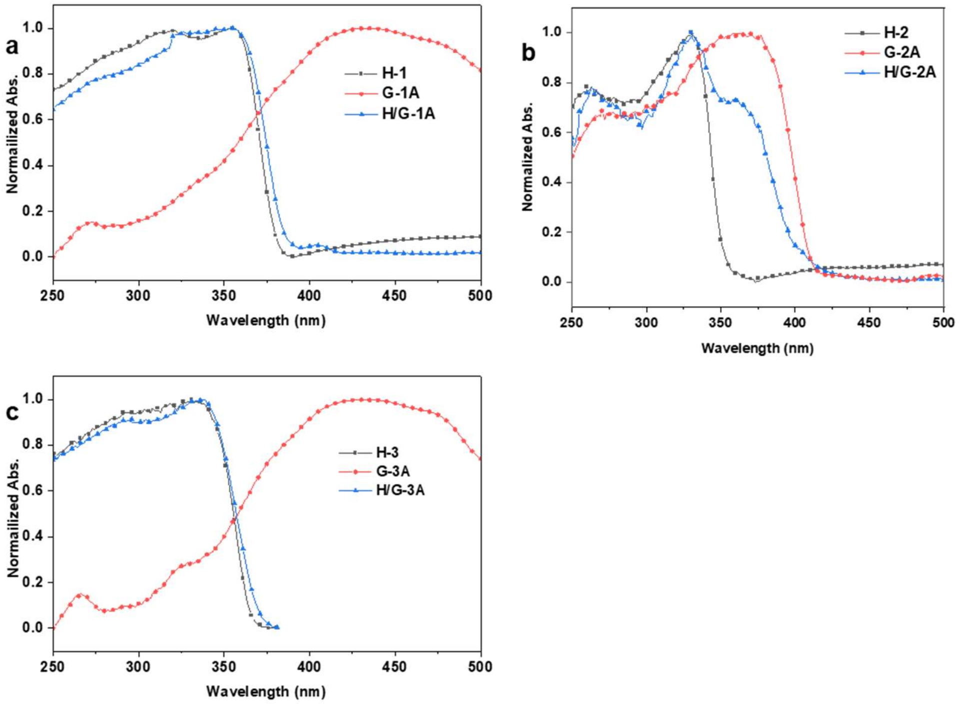


**Figure S7.** Solid-state UV-vis spectra of H-1, G-1A and H/G-1A (a); H-2, G-2A and H/G-2A (b); H-3, G-3A, H/G-3A (c).

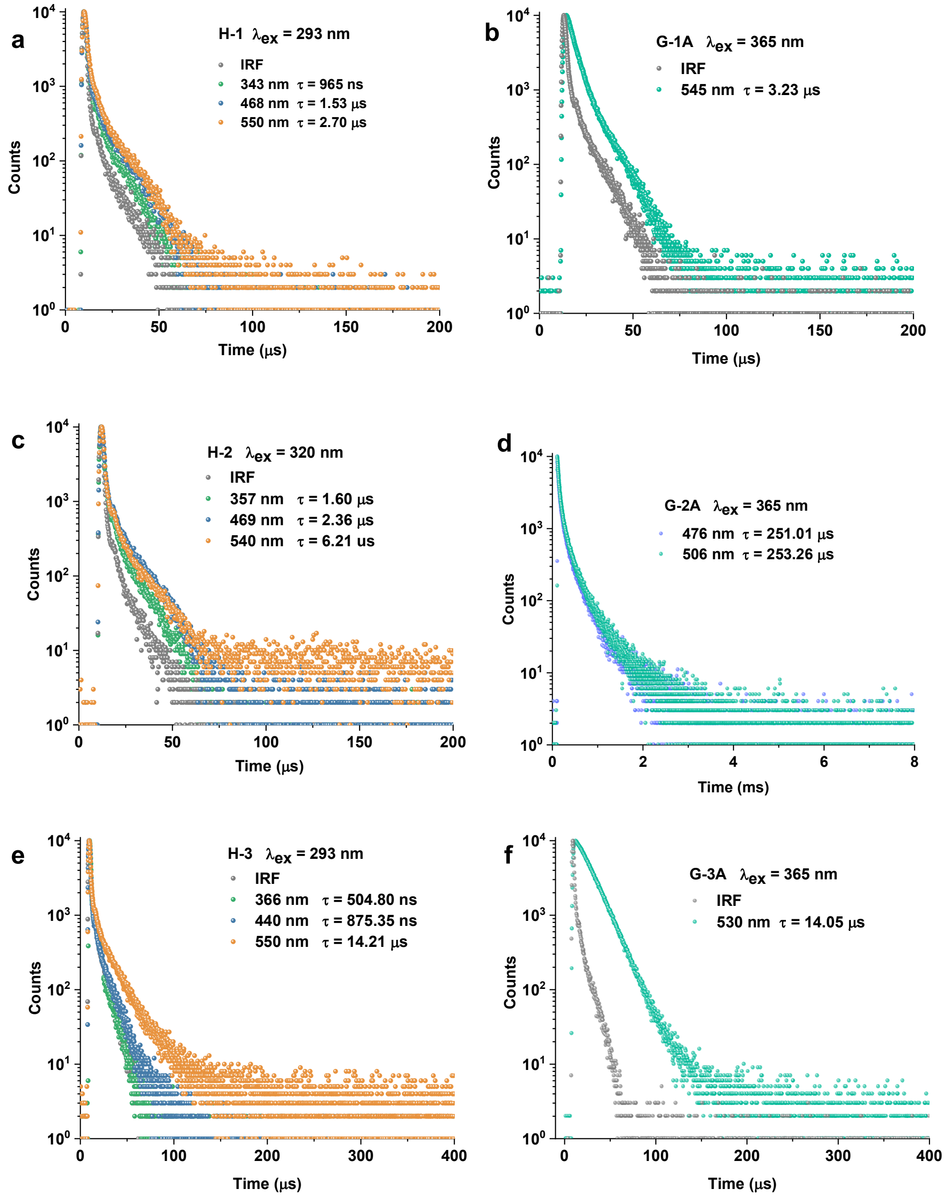


**Figure S8.** (a) PL decay curves of H-1 excited at 293 nm. (b) PL decay curves of G-1A excited at 365 nm. (c) PL decay curves of H-2 excited at 320 nm. (d) PL decay curves of G-2A excited at 365 nm. (e) PL decay curves of H-3 excited at 293 nm. (f) PL decay curves of G-2A excited at 365 nm.

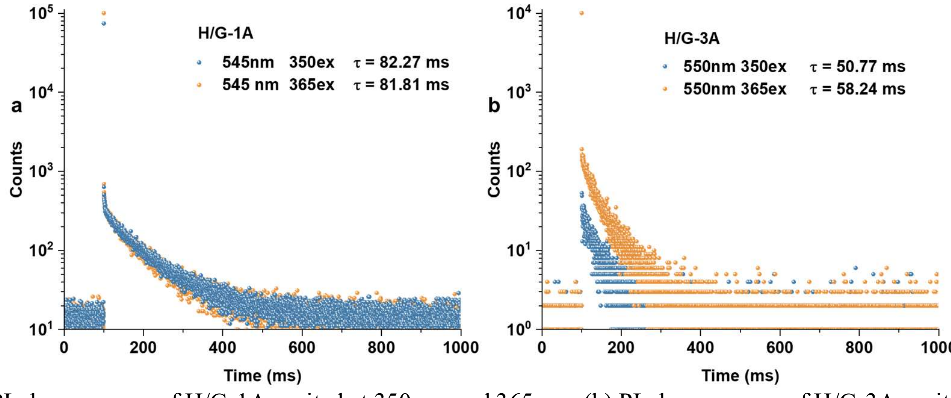


**Figure S9.** (a) PL decay curves of H/G-1A excited at 350 nm and 365 nm. (b) PL decay curves of H/G-3A excited at 350 nm and 365 nm.

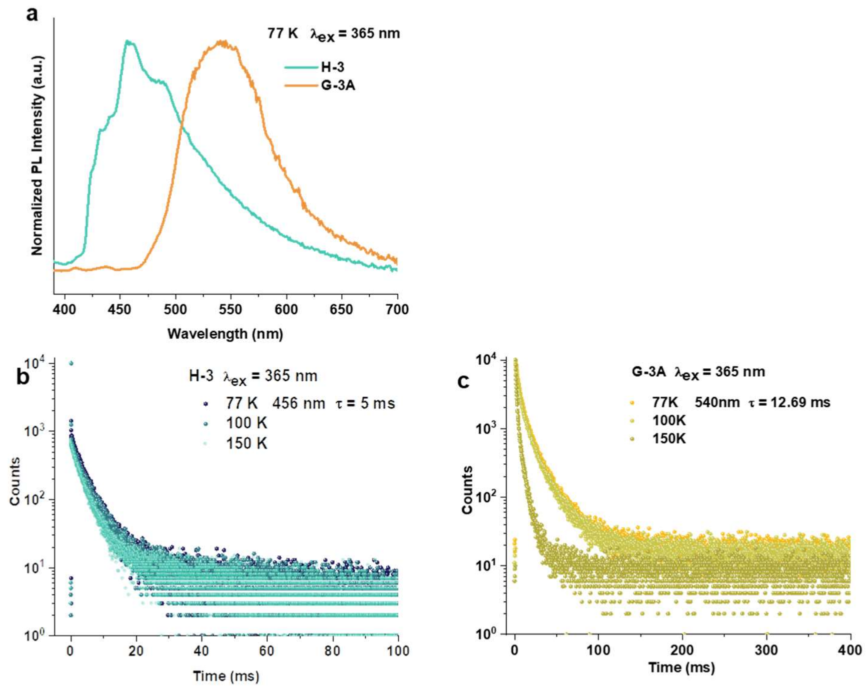


**Figure S10.** (a) Normalized steady state PL spectra of H-3 and G-3A at 77 K. (b) PL decay curves of H-3 at 77 K, 100 K and 150 K. (c) PL decay curves of G-3A at 77 K, 100 K and 150 K.

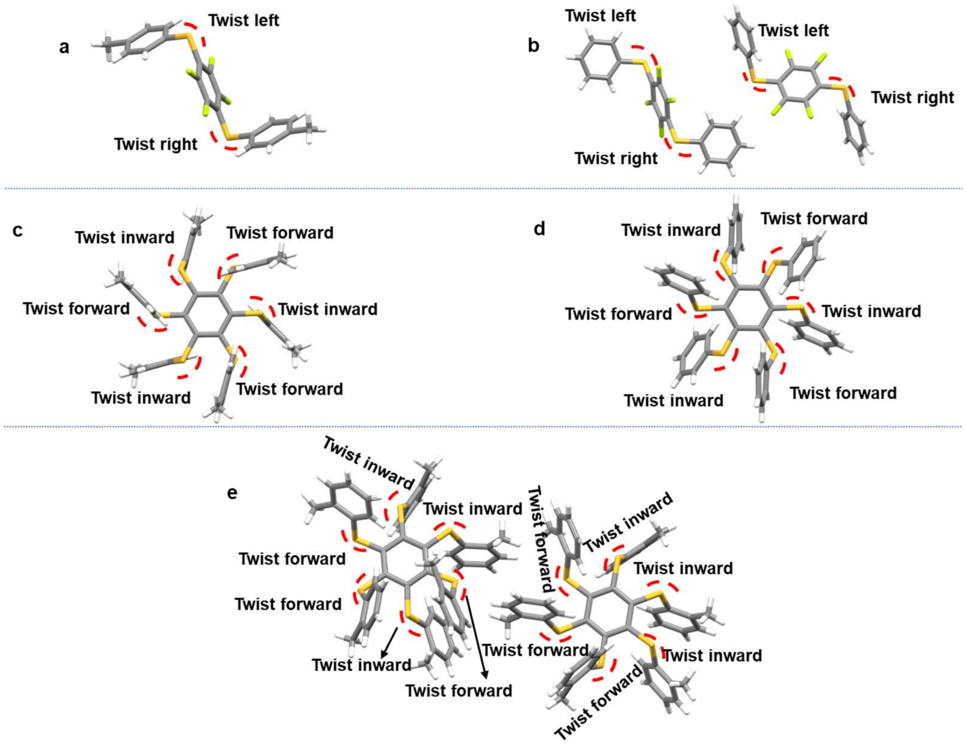


**Figure S11.** Single crystal conformation of H-1 (a); H-3 (b); G-1A (c); G-3A (d); G-1B (e) extracted from single crystal data.

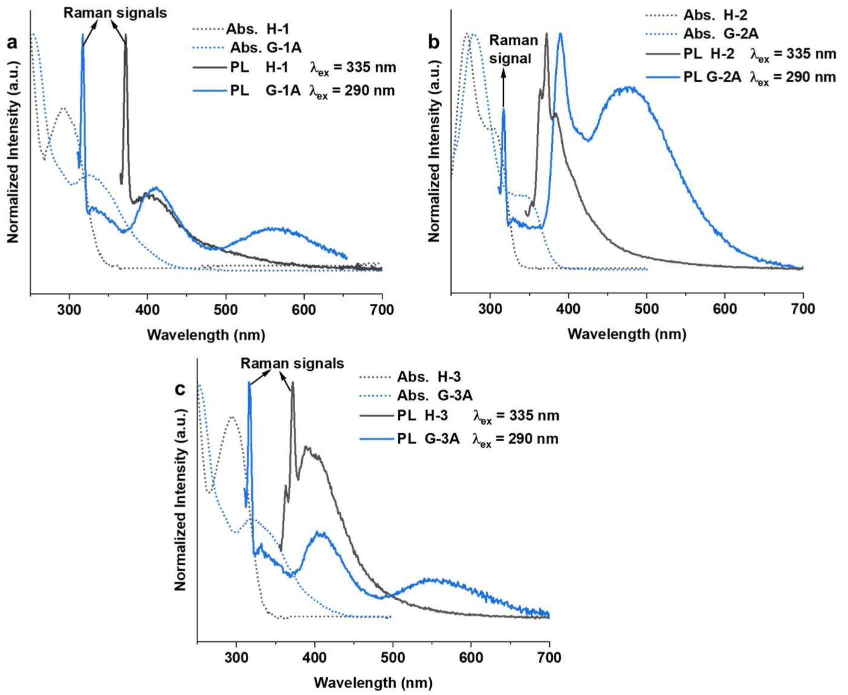


**Figure S12.** Normalized UV absorption and steady state fluorescence spectra of (a)H/G-1A; (b) H/G-2A; (c) H/G-3A in ACN solution (10 μM).

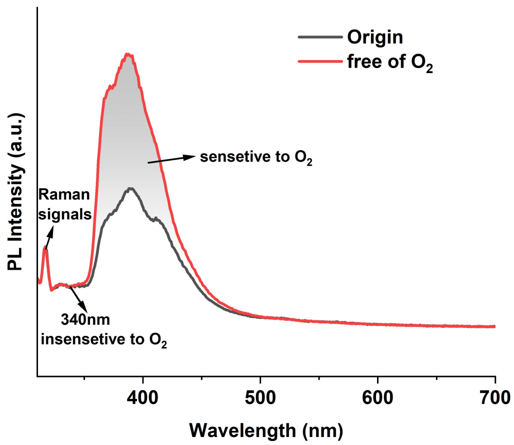


**Figure S13.** PL spectrum of H/G-2A in ACN (10 μM) before and after deoxygenation by bubbling with $N_2$.

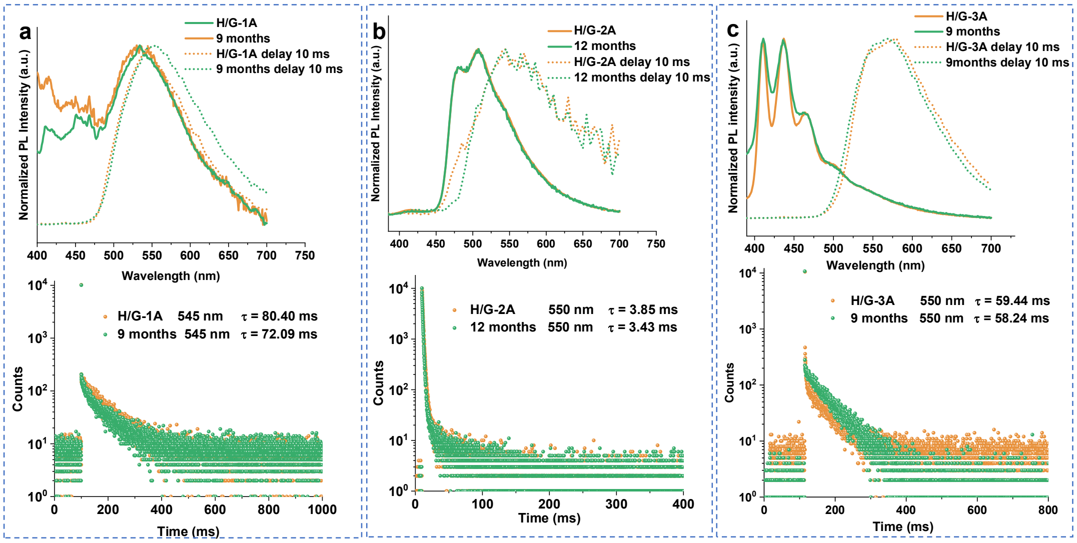


**Figure S14.** Steady state PL spectra and corresponding decay curves of H/G-1A before and after placing 12 months (a); H/G-2A before and after placing 12 months (b); H/G-3A before and after placing 9 months (c).

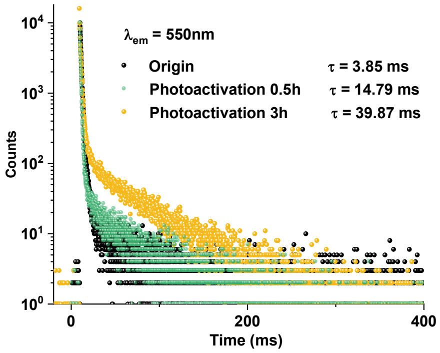


**Figure S15.** PL decay spectra of H/G-2A before and after UV (365 nm) photoactivation for 0.5 h and 3h.

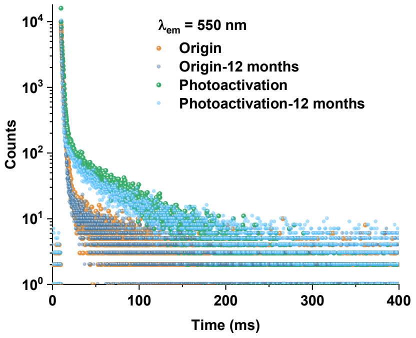


**Figure S16.** PL decay spectra of H/G-2A before and after photoactivation for 3h, as well as placing in ambient condition for 12 months.

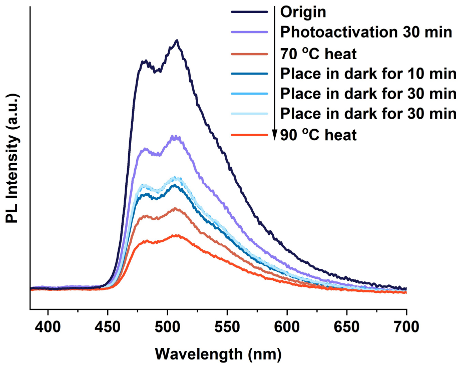


**Figure S17.** Steady state PL spectra of H/G-2A before and after photoactivation (365 nm), followed by heating treatment and placing in dark at room temperature.

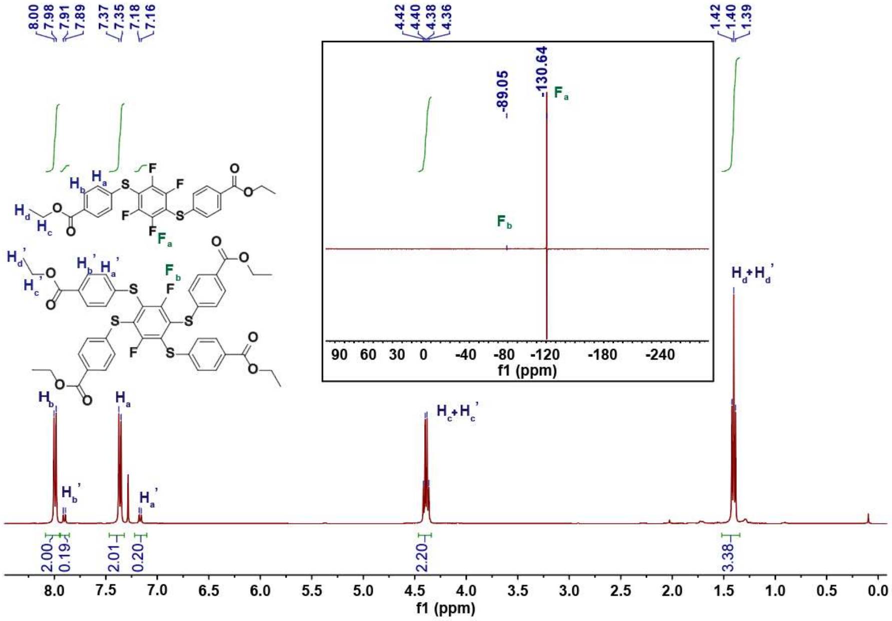


**Figure S18.** $^{1}$H NMR spectra of H/G-2A after photoactivation for 3 h with the doping ratio of 1: 5% calculated based on the integrated areas. Insert: $^{19}$F NMR spectra of H/G-2A after photoactivation for 3 h.

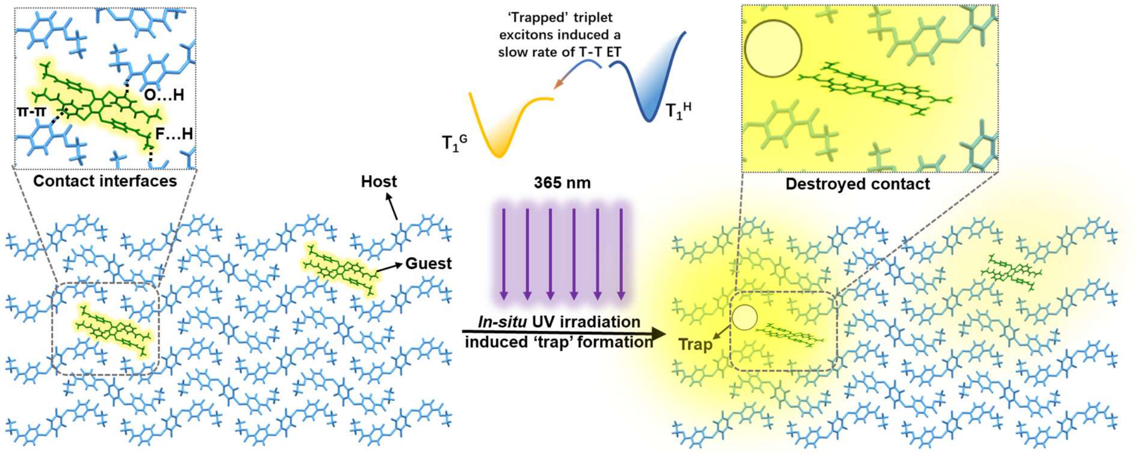


**Figure S19.** Proposed scheme of *in-situ* UV irradiation induced 'trap' formation based on physical viewpoints.

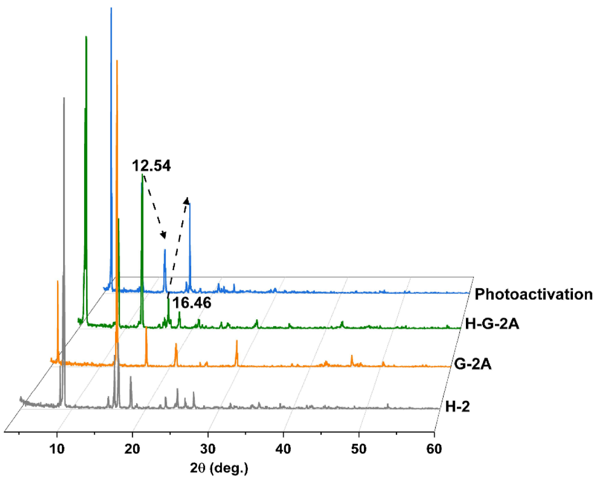


**Figure S20.** PXRD of crystalline H-2, G-2A, H/G-2A and H/G-2 after photoactivation for 3 h.

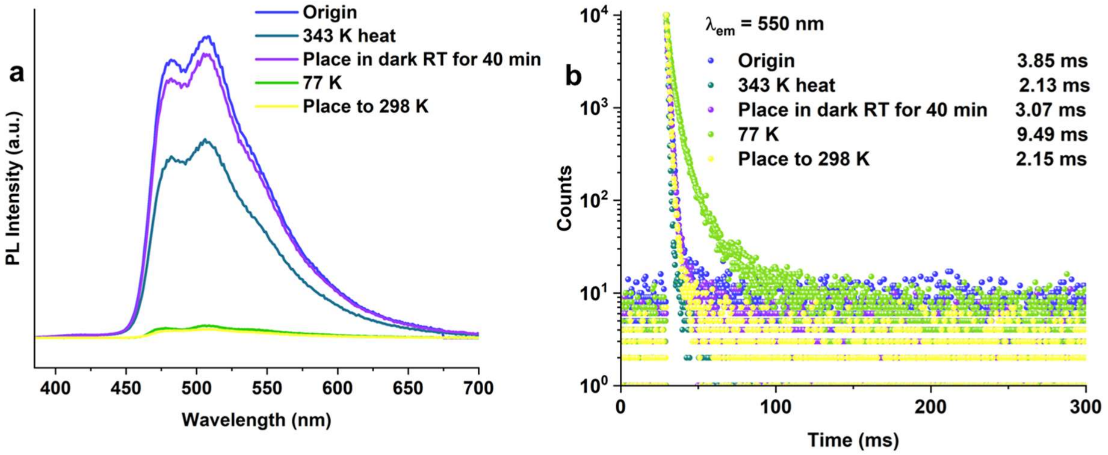


**Figure S21.** Steady state PL spectra and corresponding decay curves of H/G-2A before and after heating and cooling. RT: room temperature.

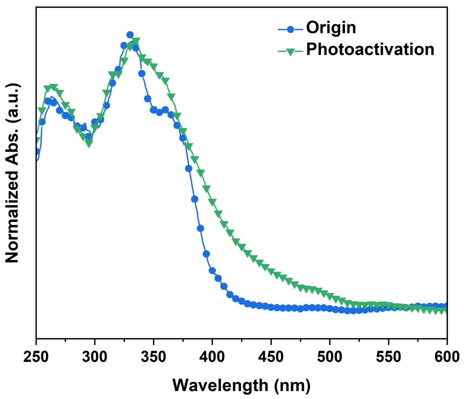


**Figure S22.** Solid-state UV spectra of crystalline H/G-2A before and after photoactivation.

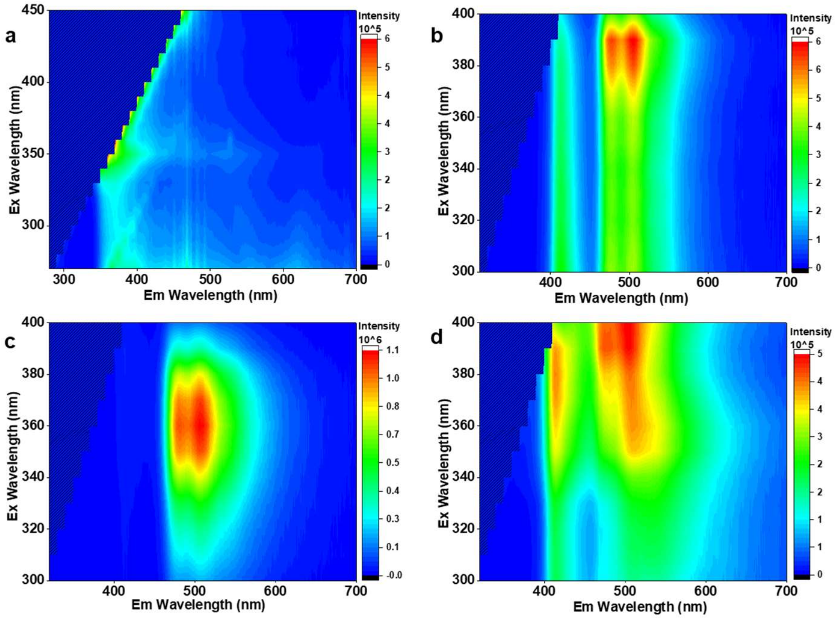

**Figure S23.** Excitation-emission mapping of crystalline H-2 (a); G-2A (b); H/G-2A before(c) and after (d) photoactivation.

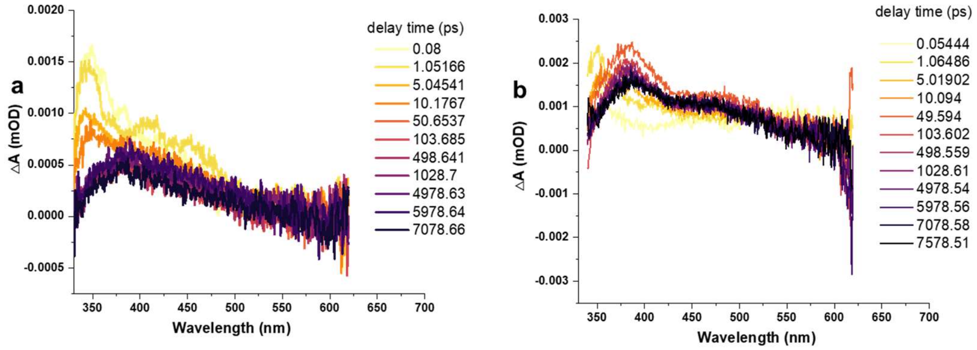

**Figure S24.** Transient absorption spectra of H/G-2A before (a) and after (b) photoactivation in the state of crystals films under 320 excitations.

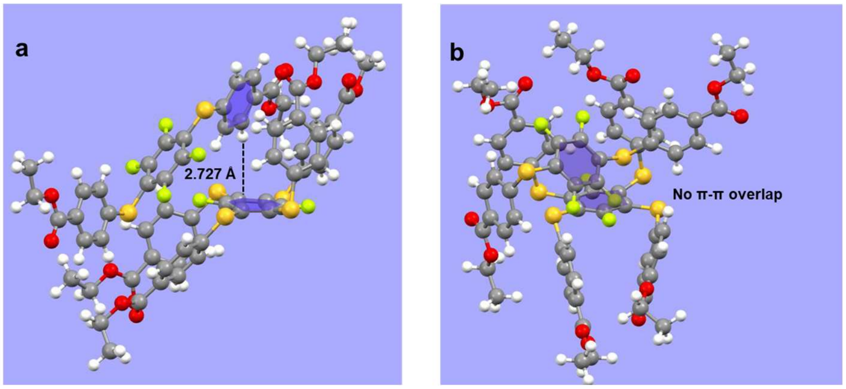

**Figure S25.** Optimized conformers of H/G-2A conf 1 (a) and H/G-2A conf 2 (b) to the ground state.

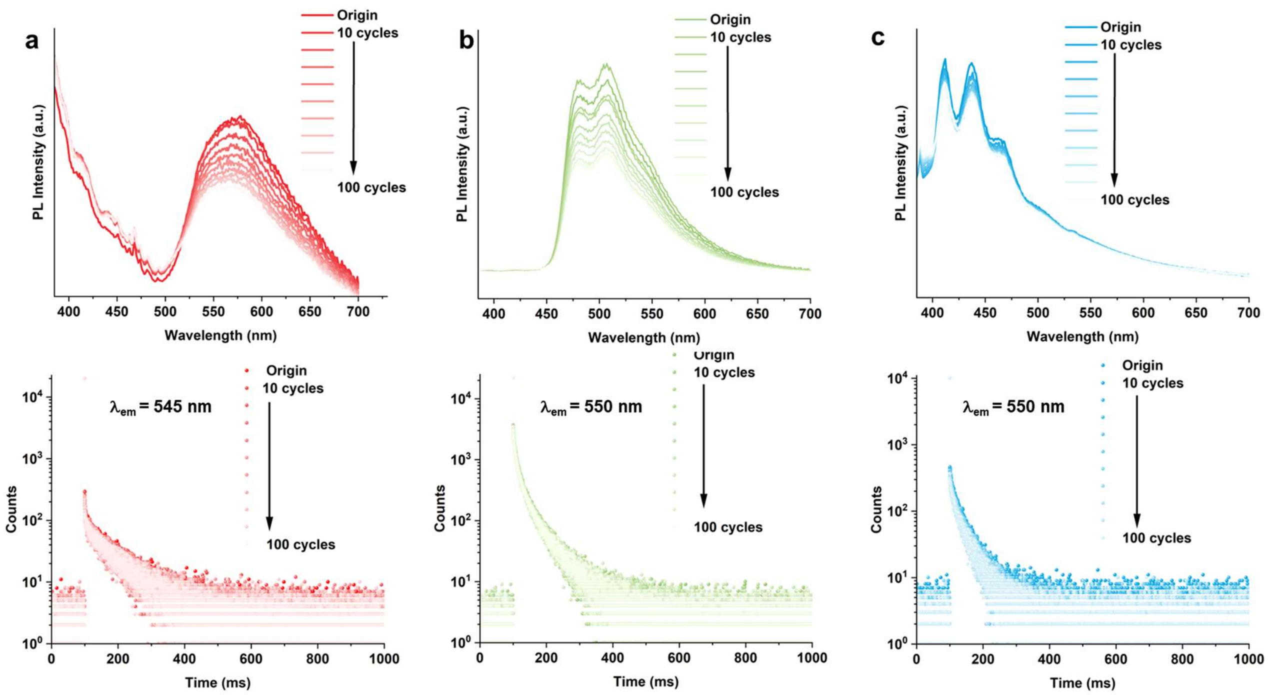


**Figure S26.** Steady state PL spectra and corresponding decay curves ($\lambda_{ex}$ = 365 nm) of (a) H/G-1A; (b) H/G-2A; (3) H/G-3A after photoirradiation (365 nm) of UV lamp for 10 cycles to 100 cycles. Each cycle is performed by UV irradiation for 0.5 min, then followed by standing for 1 min.

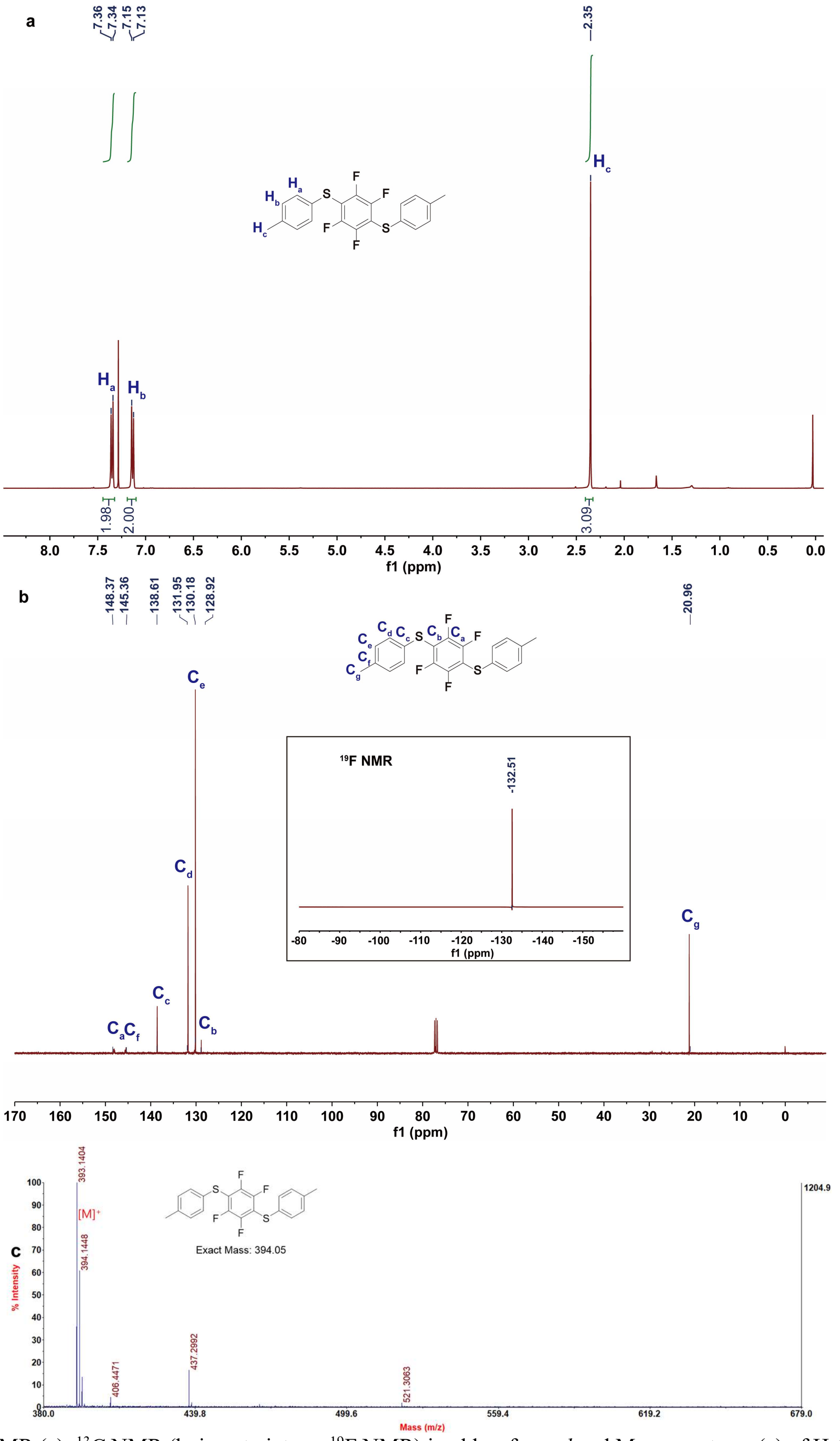


**Figure S27.** $^{1}$H NMR (a), $^{13}$C NMR (b, insert picture: $^{19}$F NMR) in chloroform-*d* and Mass spectrum (c) of H-1.

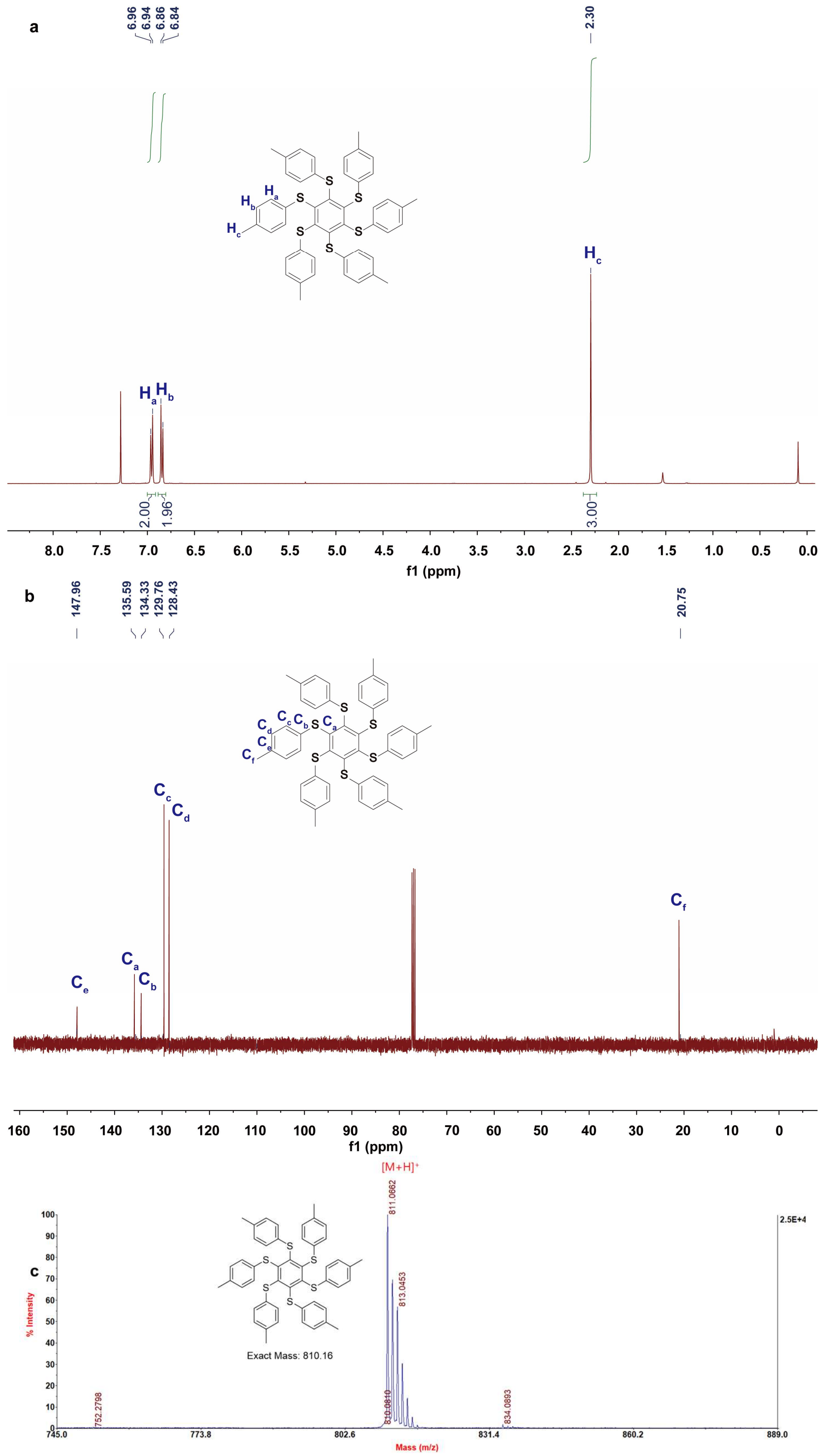


**Figure S28.** $^{1}$H NMR (a), $^{13}$C NMR spectra (b) of G-1A in chloroform-*d* and MALDI-TOF mass spectrum (c) of G-1A.

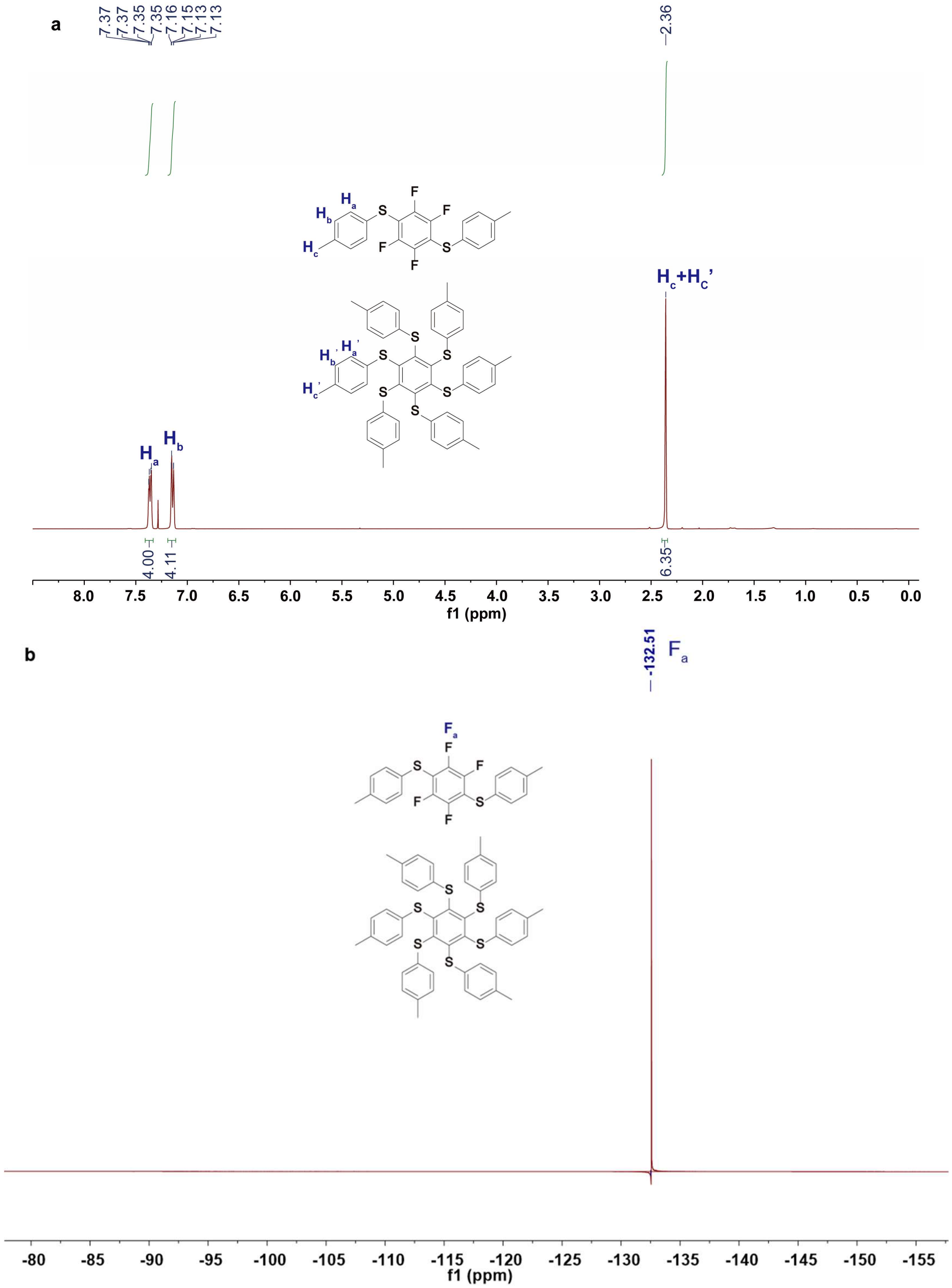


**Figure S29.** $^{1}$H NMR (a), $^{19}$F NMR spectra (b) of H/G-1A in chloroform-*d*.

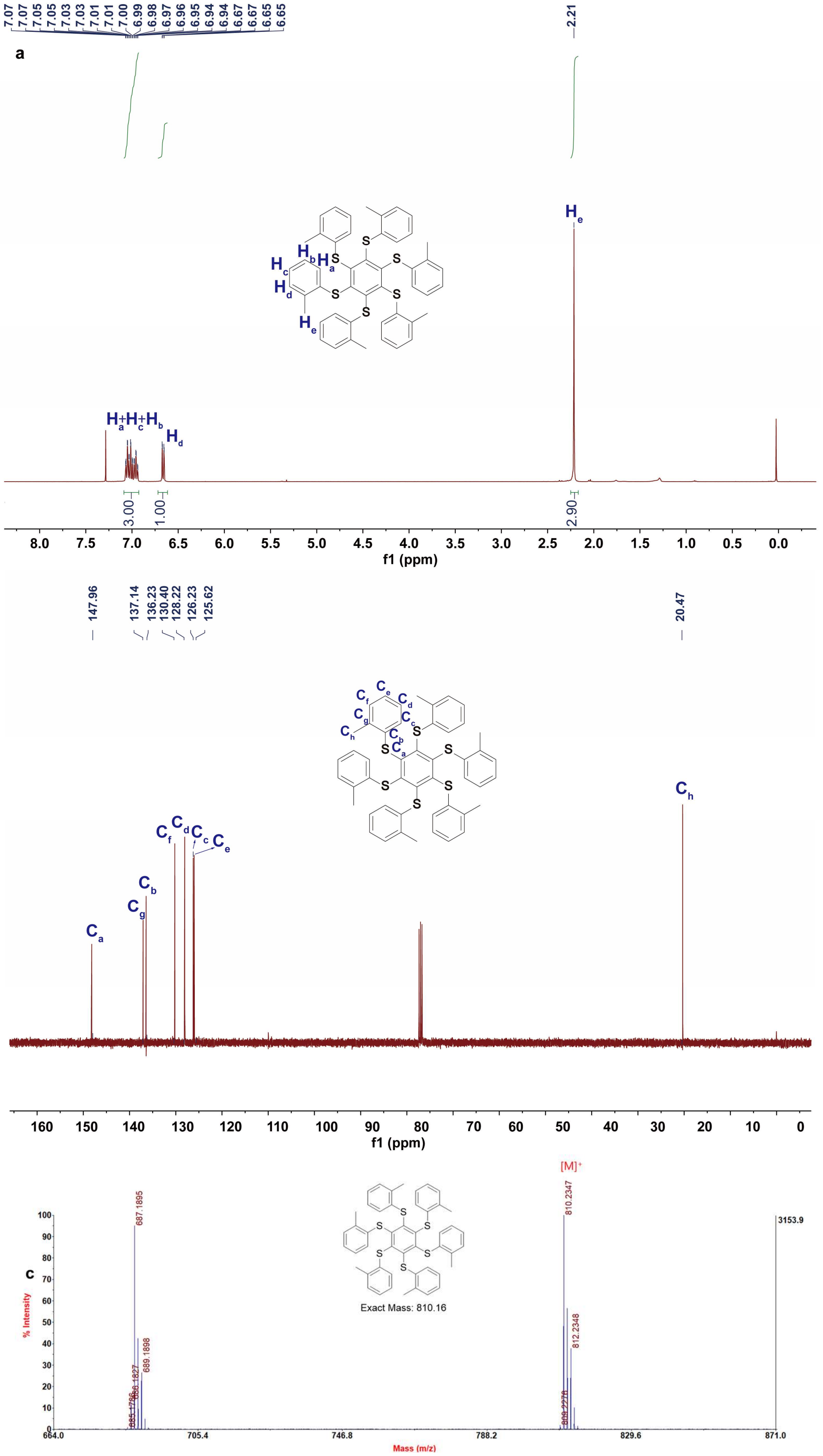


**Figure S30.** $^{1}$H NMR (a), $^{13}$C NMR spectra (b) of G-1B in chloroform-*d* and MALDI-TOF mass spectrum (c) of G-1B.

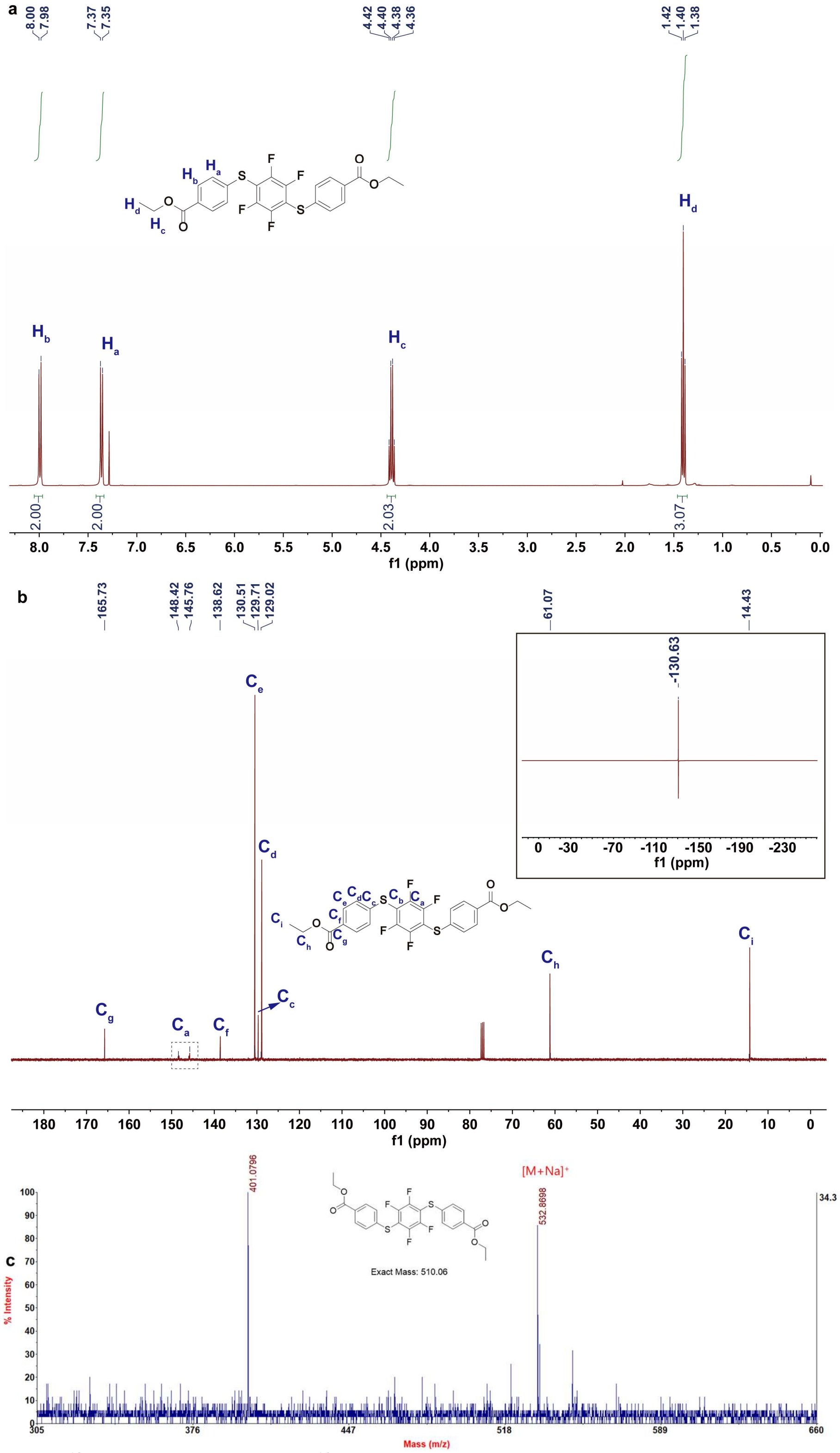


**Figure S31.** $^{1}H$ NMR (a), $^{13}C$ NMR (b, inset picture: $^{19}F$ NMR) spectra of H-2 in chloroform-*d* and MALDI-TOF mass spectrum (c) of H-2.

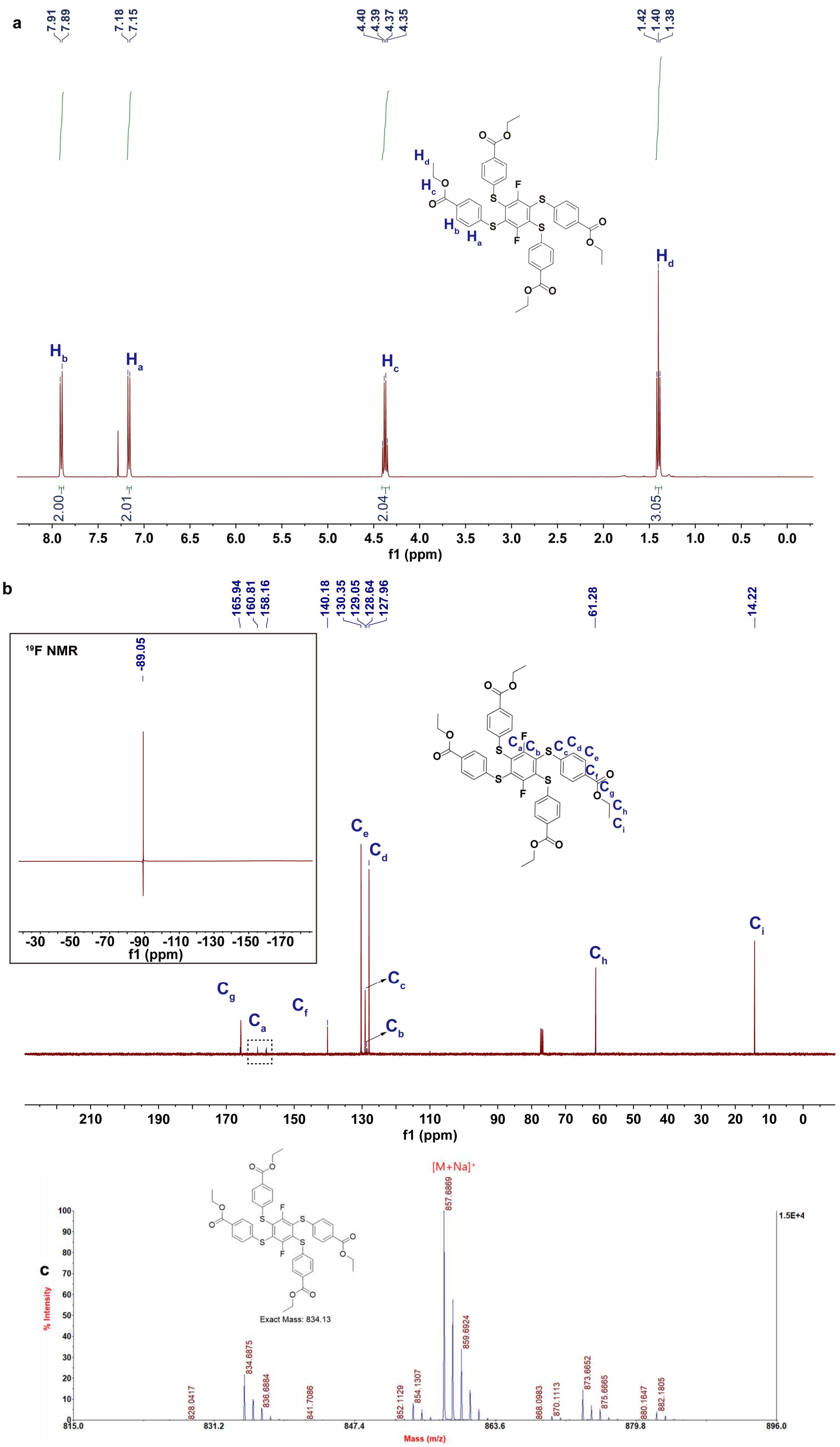


**Figure S32.** $^{1}H$ NMR (a), $^{13}C$ NMR (b, inset picture: $^{19}F$ NMR) spectra of G-2A in chloroform-*d* and MALDI-TOF mass spectrum (c) of G-2A.

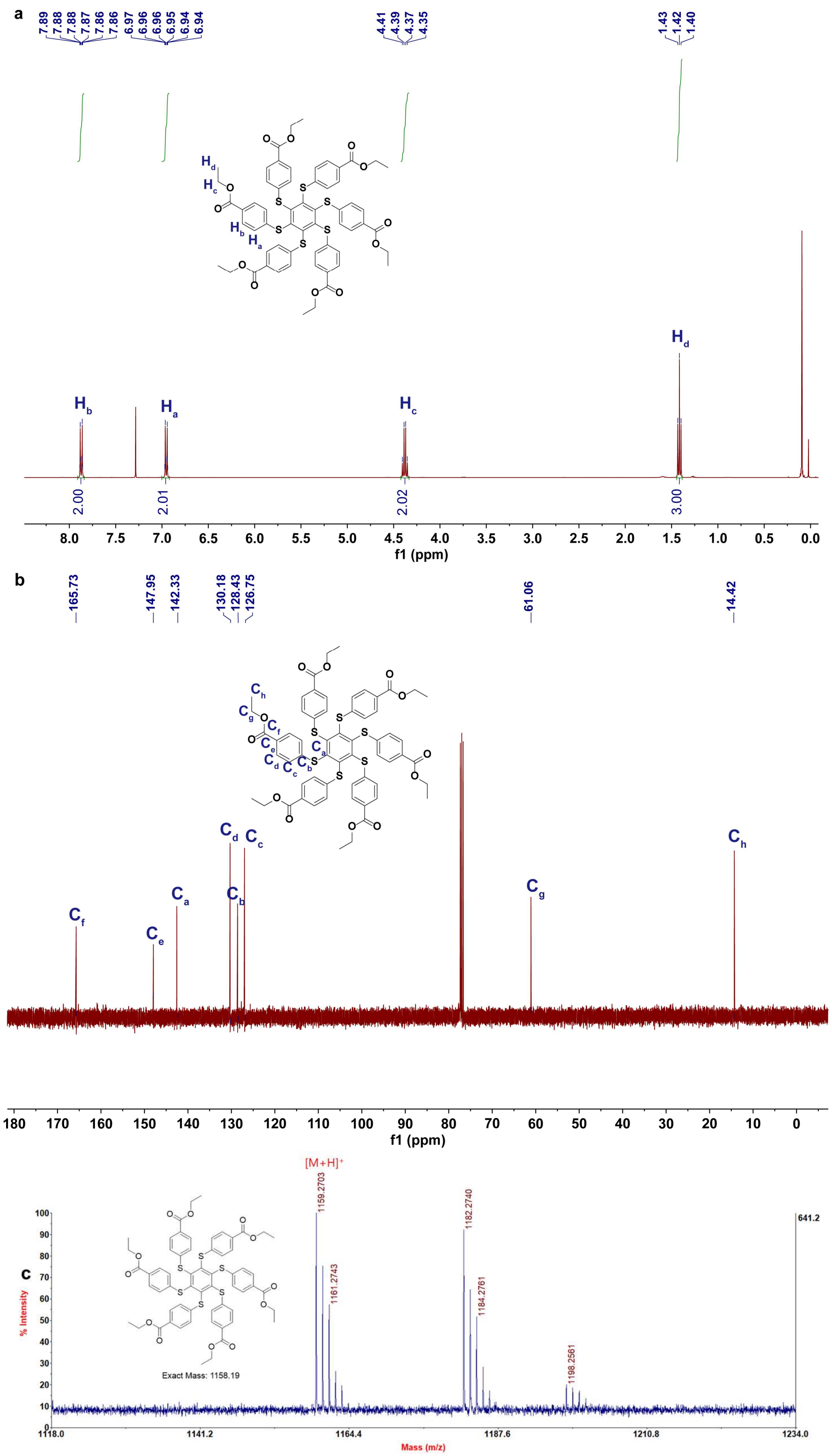


**Figure S33.** (a) $^{1}H$ NMR, (b) $^{13}C$ NMR spectra of G-2B in chloroform-*d* and (c) MALDI-TOF mass spectrum of G-2B.

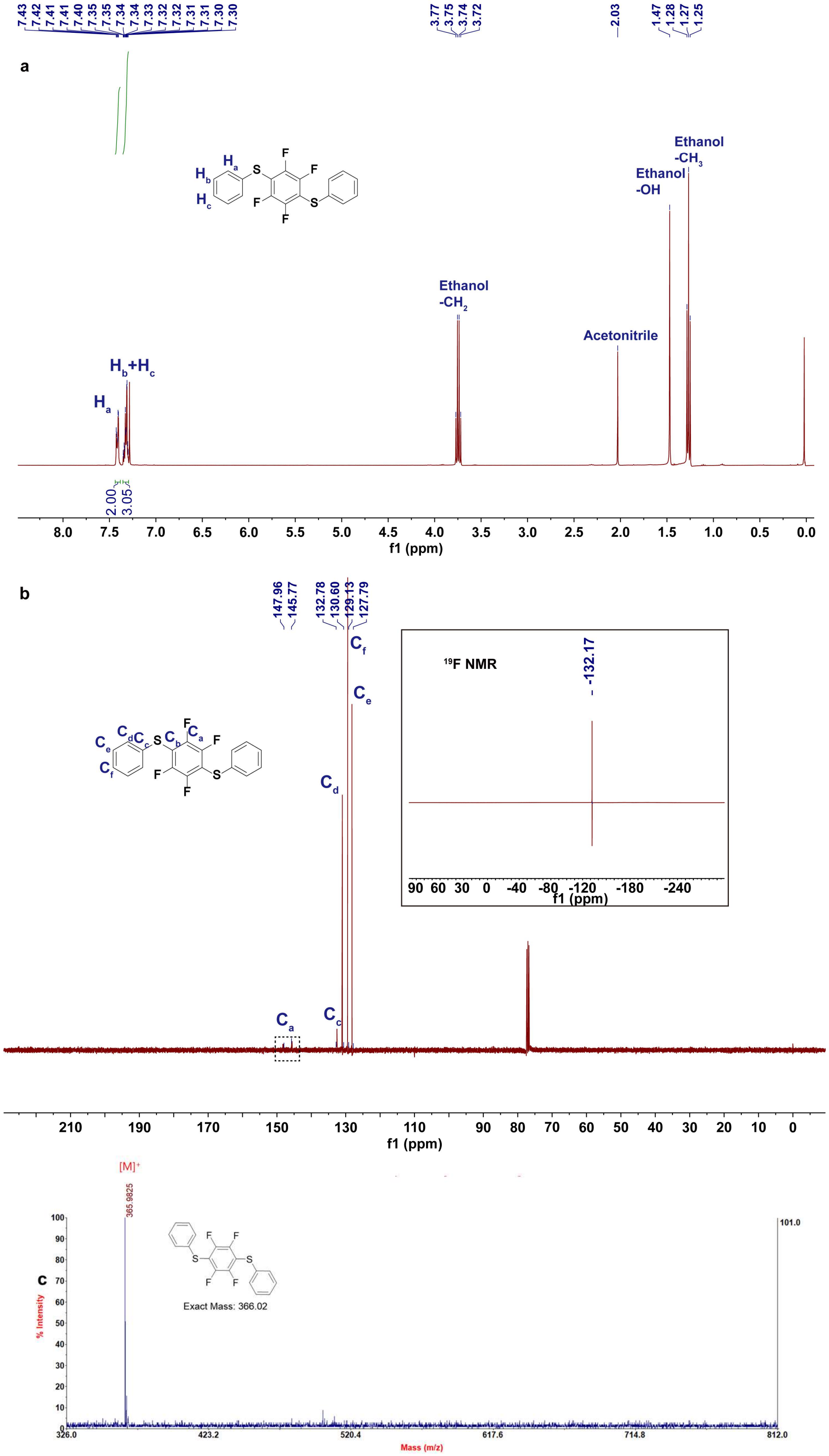


**Figure S34.** (a) $^{1}$H NMR (with residual solvent), (b) $^{13}$C NMR spectra (inset picture: $^{19}$F NMR) of H-3 in chloroform-*d* and (c) MALDI-TOF mass spectrum of H-3.

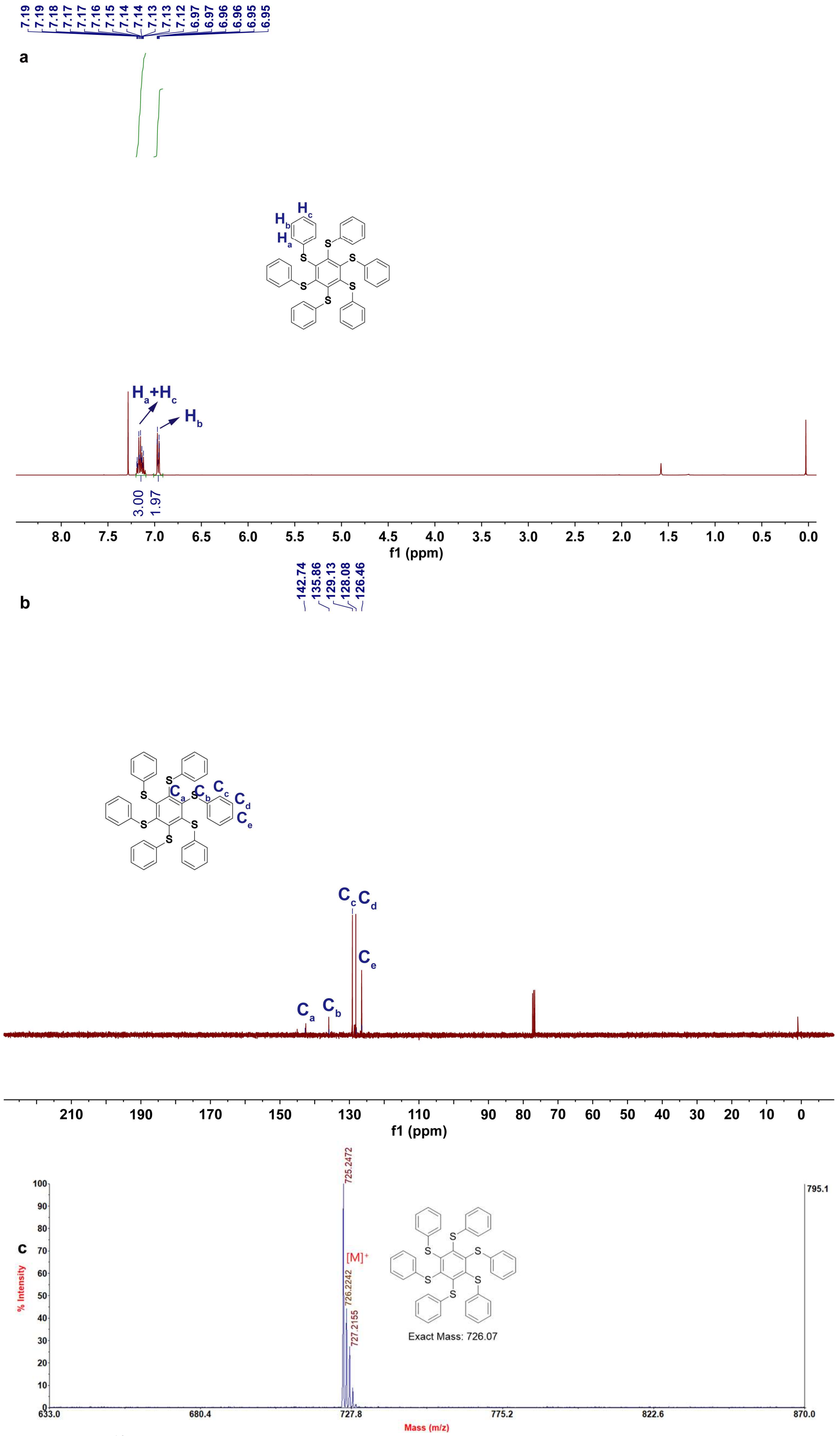


**Figure S35.** (a) $^{1}$H NMR, (b) $^{13}$C NMR spectra of G-3A in chloroform-*d* and (c) MALDI-TOF mass spectrum of G-3A.

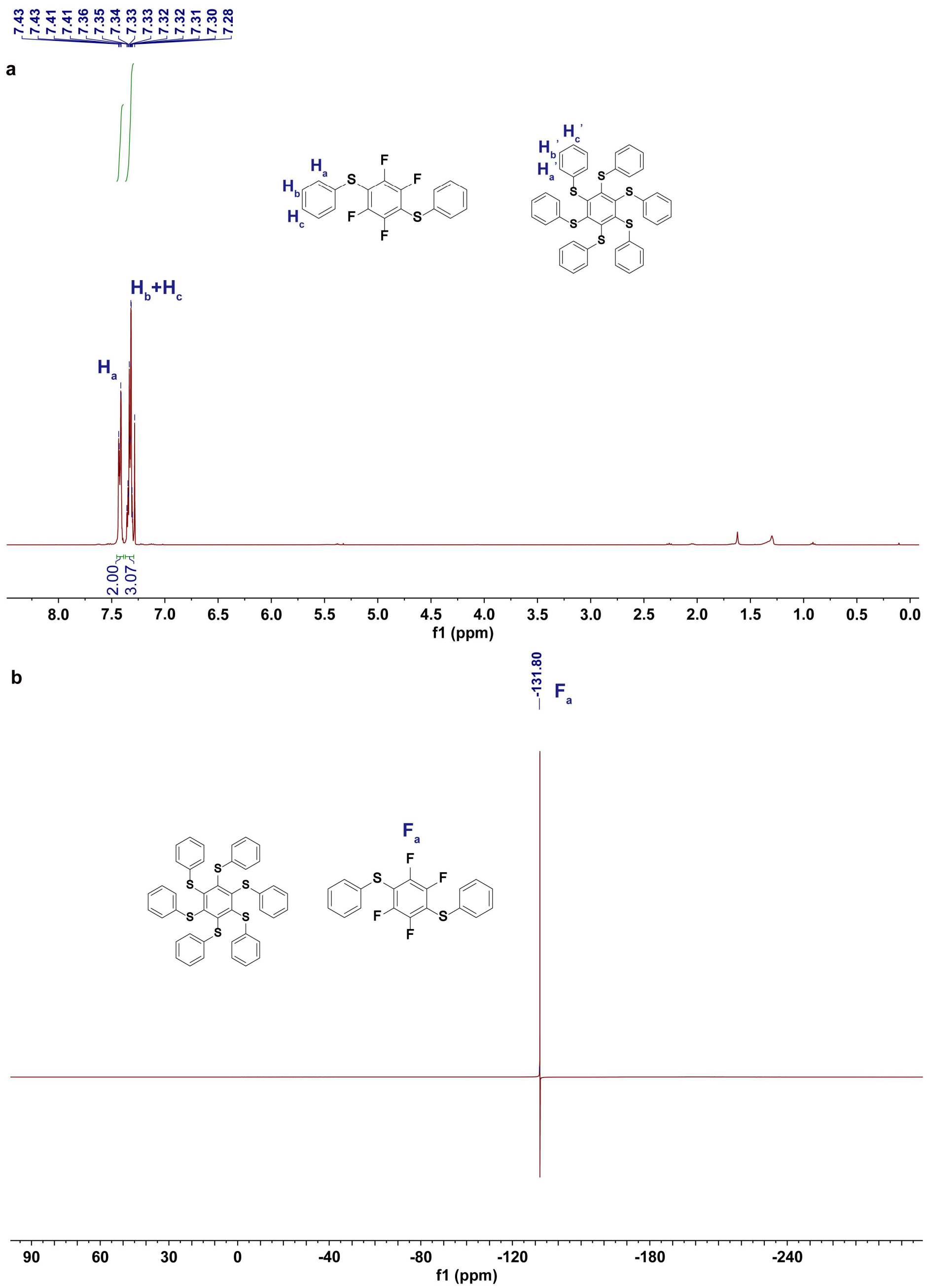


**Figure S36.** $^{1}$H NMR (a), $^{19}$F NMR (b) spectra of H/G-3A in chloroform-*d*.

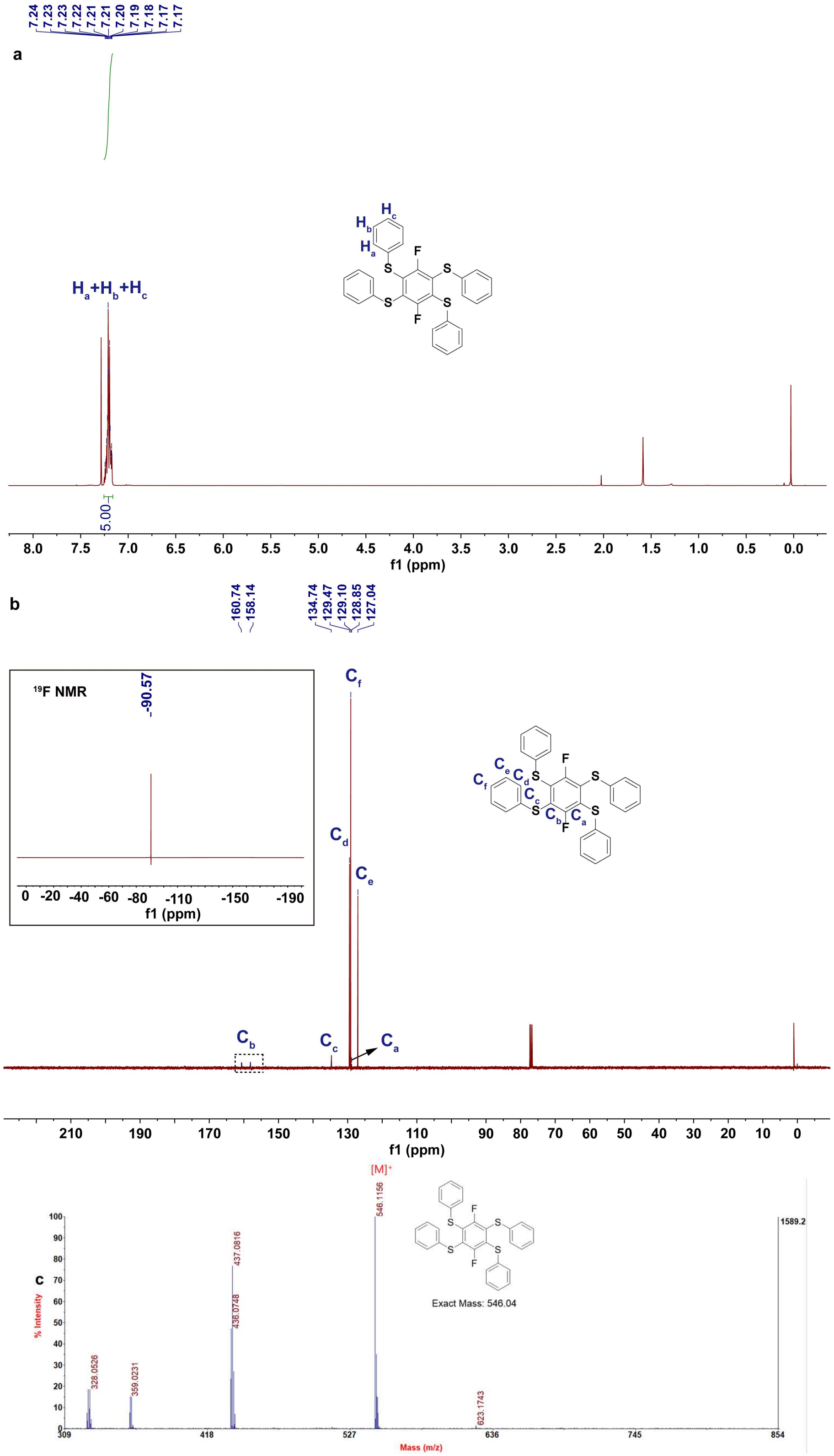


**Figure S37.** $^{1}$H NMR(a), $^{13}$C NMR (b, inset picture: $^{19}$F NMR) spectra of G-3B in chloroform-*d* and MALDI-TOF mass spectrum (c) of G-3B.

## Supporting Tables

**Table S1.** Photoluminescence property of the cross-doped H/G crystals

| Compound | H-1 | H-2 | H-3 |
|---|---|---|---|
| G-1A | τ>10ms | 1ms<τ<5ms | 1ms<τ<10ms |
| G-1B | No pRTP | No pRTP | No pRTP |
| G-2A | No pRTP | 1ms<τ<10ms | No pRTP |
| G-2B | No pRTP | No pRTP | No pRTP |
| G-3A | 1ms<τ<10ms | 1ms<τ<10ms | τ>10ms |
| G-3B | No pRTP | No pRTP | No pRTP |

**Table S2.** TD-DFT data for individual hosts and guests species, where the host and guest conformations are extracted from single crystal data.

| | $E(S_0\text{-}S_n)_{abs.}$, nm | $E(S_1\text{-}S_0)_{flu.}$, nm | $E(T_1\text{-}S_0)_{phos.}$ at $T_1$, nm | $E(T_1\text{-}S_0)_{phos.}$ at $S_1$, nm | $\langle S_1\vert\hat{H}_{SO}\vert T_1\rangle$, $cm^{-1}$ | $k_{ISC}$, $s^{-1}$ | $k_{flu.}$, $s^{-1}$ | $k_{phos.}$ (at $S_1/T_1$)$s^{-1}$ |
|---|---|---|---|---|---|---|---|---|
| H-2 | 320 (n=1) $(330)^{exp}$ | 393 $(370)^{exp}$ | 858 | 421 | 5.08 | $4.4\times10^{10}$ | $6\times10^5$ | $2\times10^4/2\times10^2$ |
| G-2A | 371 (n=1) $(375)^{exp}$ | 395 $(410)^{exp}$ | 527 $(476)^{exp}$ | 448 | 9.74 | $2.7\times10^{10}$ | $5\times10^7$ | $5\times10^2/7\times10^1$ $(1\times10^4)$ |
| G-2B | 503 (n=1) 470 (n=2) 410 (n=3) 397 (n=4) 394 (n=5) $(400\text{-}475)^{exp}$ | 492 | 563 $(554)^{exp}$ | 514 | 2.28 | $1.9\times10^{10}$ | $5\times10^6$ | $6\times10^2/7\times10^2$ $(4\times10^5)$ |
| H-3 | 298 (n=1) $(327)^{exp}$ | 418 $(388)^{exp}$ | 907 | 441 | 0.37 | $3.4\times10^8$ | $6\times10^4$ | $1\times10^2/2\times10^3$ |
| G-3A | 409 (n=1,2) 351 (n=3) 342 (n=4,5) $(400\text{-}475)^{exp}$ | 503 | 2033 | 527 $(530)^{exp}$ | 1.73 | $1.1\times10^{10}$ | $8\times10^5$ | $1\times10^3/8\times10^1$ $(2\times10^5)$ |
| G-3B | 356 (n=1) $(375)^{exp}$ | 460 $(425)^{exp}$ | 772 | 479 $(476)^{exp}$ | 2.23 | $1.7\times10^{10}$ | $2\times10^5$ | $8\times10^1/7\times10^2$ $(1.6\times10^5)$ |

**Table S3.** Natural transition orbitals (NTOs) and corresponding weights for $S_1$ and $T_1$ states of compounds H-2, G-2A, G-2B and their mixed dimers calculated at $S_1$ and $T_1$ state geometry.

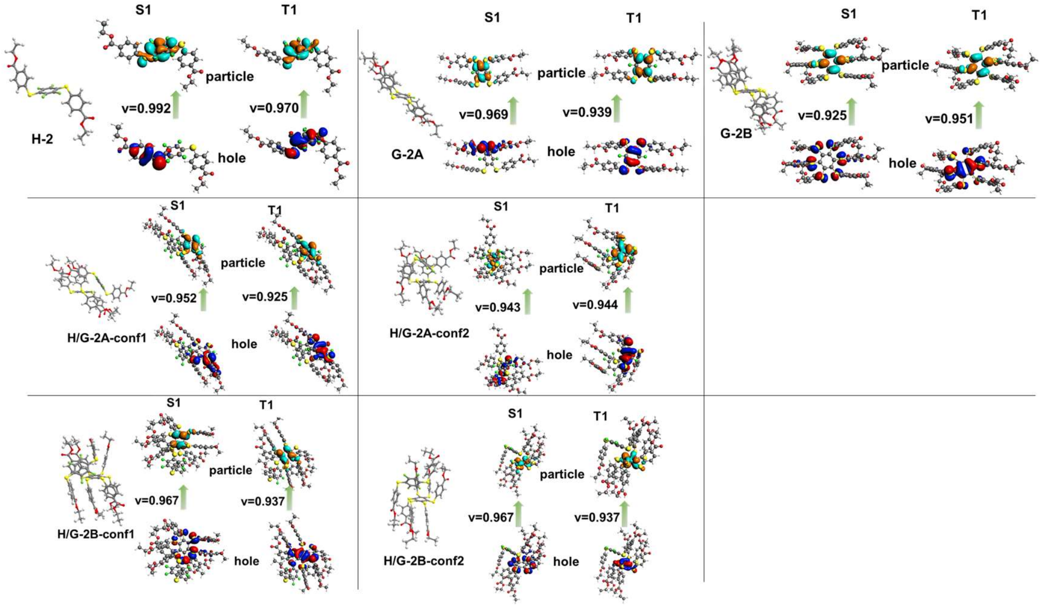


**Table S4.** NTOs and corresponding weights for $S_1$ and $T_1$ states of compounds H-3, G-3A, G-3B and their mixed dimers calculated at $S_1$ and $T_1$ state geometry.

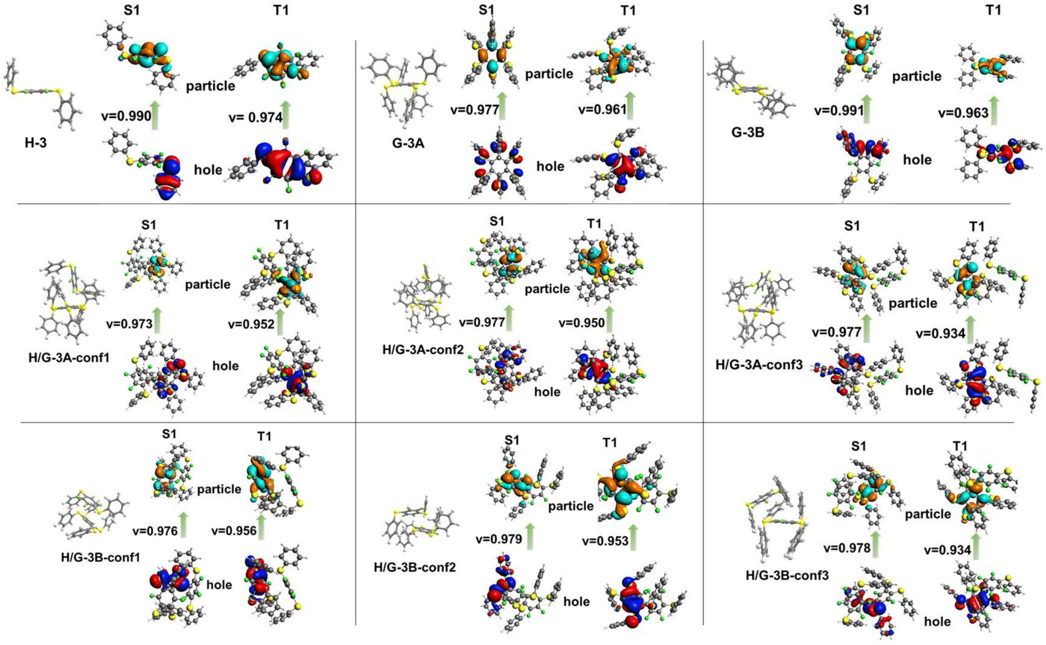

**Table S5.** Calculated conformations of H-1, H-3, G-1A and G-3A at $S_0$, $S_1$ and $T_1$ state geometry.

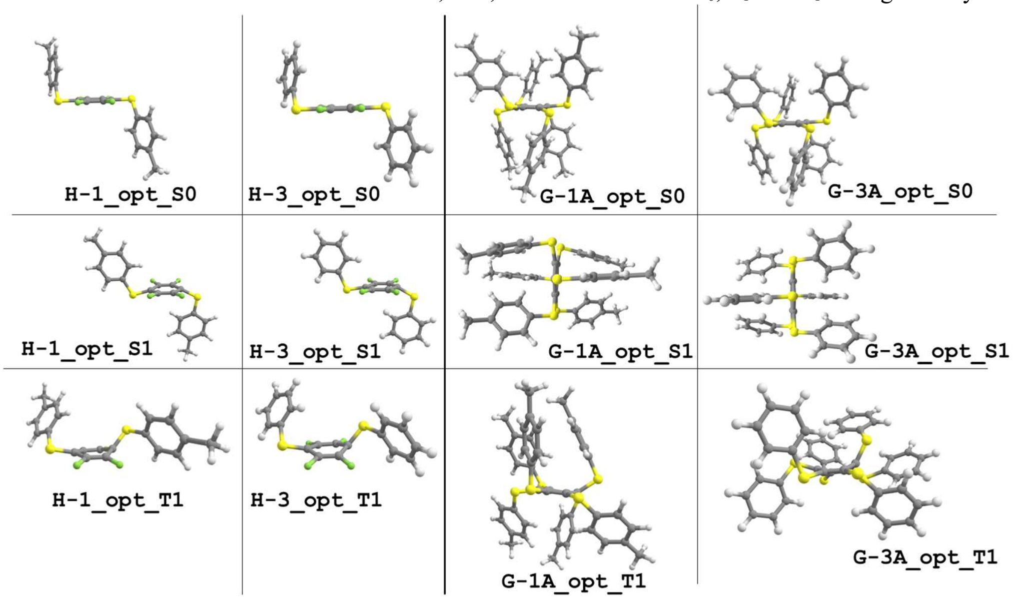

**Table S6.** Dimer complexes of hosts (H-2, H-3) with guests (G-2A, G-2B, G-3A, G-3B) in different conformations, where the individual host and guest conformations are extracted from single crystal data.

| | $E(S_1\text{-}S_0)_{flu.}$, nm | $E(T_1\text{-}S_0)_{phos.}$ at $T_1$, nm | $E(T_1\text{-}S_0)_{phos.}$ at $S_1$, nm | $\langle S_1 \vert \hat{H}_{SO} \vert T_n \rangle$, $cm^{-1}$ | $k_{ISC}$, $s^{-1}$ | $k_{flu.}$, $s^{-1}$ | $k_{phos.}$ (at $S_1/T_1$) $s^{-1}$ |
|---|---|---|---|---|---|---|---|
| H/G-2A conf1 | 374 | 571 | 444 (475)$^{exp}$ | 11.96 | $5.0\times10^{9}$ | $6\times10^{7}$ | $7\times10^{2}/4\times10^{1}$ |
| H/G-2A conf2 | 376 | 713 | 513 (475)$^{exp}$ | 3.60 (n=1)<br>3.06 (n=2) | $2.2\times10^{8}$<br>$4.6\times10^{10}$ | $1\times10^{8}$ | $1\times10^{2}/7\times10^{1}$ |
| H/G-2B conf1 | 461 | 561 | 484 | 5.09 | $7.7\times10^{10}$ | $4\times10^{6}$ | $1\times10^{3}/3\times10^{2}$ |
| H/G-2B conf2 | 453 | 772 | 483 | 6.44 | $6.0\times10^{10}$ | $1\times10^{7}$ | $5\times10^{2}/4\times10^{1}$ |
| H/G-3A conf1 | 499 | 996 | 517 (560)$^{exp}$ | 2.96 | $3.4\times10^{10}$ | $4\times10^{5}$ | $7\times10^{2}/8\times10^{2}$ |
| H/G-3A conf2 | 491 | 1014 | 511 (560)$^{exp}$ | 1.51 | $3.4\times10^{9}$ | $3\times10^{5}$ | $1\times10^{4}/1\times10^{2}$ |
| H/G-3A conf3 | 486 | 672 | 510 (560)$^{exp}$ | 3.92 | $5.0\times10^{10}$ | $5\times10^{5}$ | $4\times10^{2}/1\times10^{2}$ |
| H/G-3B conf1 | 428 | 892 | 453 (456)$^{exp}$ | 6.46 | $1.0\times10^{11}$ | $7\times10^{6}$ | $2\times10^{3}/8\times10^{1}$ |
| H/G-3B conf2 | 430 | 621 | 448 (456)$^{exp}$ | 1.07 | $3.9\times10^{9}$ | $5\times10^{5}$ | $8\times10^{2}/1\times10^{2}$ |
| H/G-3B conf3 | 438 | 532 | 466 (456)$^{exp}$ | 7.21 | $1.2\times10^{11}$ | $8\times10^{6}$ | $4\times10^{3}/2\times10^{1}$ |

**Table S7.** Literature comparison for triplet-triplet energy gap (**$\Delta E_{T\text{-}T}$**) of pRTP systems.

| System | $\Delta E_{T\text{-}T}$ | Reference |
|---|---|---|
| Host-guest pRTP system | around 0.50 eV | 18 |
| | approximately at 0.65 eV | 19 |
| | 0.66 eV | 20 |
| | 0.66 eV | 21 |
| | 0.18-0.46 eV | *This work* |
| D-A pRTP system | 0.57-0.60 eV (crystalline state)<br>0.38-0.88 (solution state) | 22 |
| | 0.15 eV | 23 |

**Table S8.** Sing crystal data of H-1, G-1A, G-1B

| Compound | H-1 | G-1A | G-1B |
|---|---|---|---|
| CCDC no. | 2176999 | 2177000 | 2177001 |
| Formula | $C_{20} H_{14} F_4 S_2$ | $C_{48} H_{42} S_6$ | $C_{48} H_{42} S_6$ |
| Formular weight | 394.43 | 811.17 | 811.17 |
| Temperature/K | 173(2) | 173(2) | 173(2) |
| Crystal system | Monoclinic | Trigonal | Triclinic |
| Space group | $P2_1/c$ | $R\bar{3}$ | $P\bar{1}$ |
| a/Å | 15.1661(8) | 21.0773(6) | 12.4505(5) |
| b/Å | 4.8806(3) | 21.0773(6) | 14.9780(6) |
| c/Å | 11.4983(6) | 7.9829(3) | 21.6769(8) |
| α/° | 90 | 90 | 92.868(2) |
| β/° | 93.913(2) | 90 | 98.0810(10) |
| γ/° | 90 | 120 | 91.7530(10) |
| V/Å$^3$ | 849.12(8) | 3071.3(2) | 3994.3(3) |
| Z | 2 | 3 | 4 |

**Table S9.** Sing crystal data of H-2, G-2A, H/G-2A

| Compound | H-2 | G-2A | H/G-2A | H/G-2A photoactivation | G-2B |
|---|---|---|---|---|---|
| CCDC no. | 2177002 | 2177003 | 2181831 | 2182332 | 2177004 |
| Formula | $C_{24} H_{18} F_4 O_4 S_2$ | $C_{42} H_{36} F_2 O_8 S_4$ | $C_{24} H_{18} F_4 O_4 S_2$ | $C_{24} H_{18} F_4 O_4 S_2$ | $C_{60} H_{54} O_{12} S_6$ |
| Formular weight | 510.50 | 834.95 | 510.50 | 510.50 | 1159.39 |
| Temperature/K | 173(2) | 173(2) | 173(2) | 173(2) | 173(2) |
| Crystal system | Monoclinic | Triclinic | Monoclinic | Monoclinic | Monoclinic |
| Space group | $P2_1/n$ | $P\bar{1}$ | $P2_1/n$ | $P2_1/n$ | $P2_1/c$ |
| a/Å | 4.8077(2) | 5.6192(5) | 4.8071(3) | 4.8120(2) | 20.7715(12) |
| b/Å | 20.6905(9) | 8.2941(8) | 20.6990(14)) | 20.6876(10) | 6.2243(3) |
| c/Å | 11.0959(5) | 21.138(2) | 11.0964(7) | 11.0963(6) | 22.3727(12) |
| α/° | 90 | 79.178(4) | 90 | 90 | 90 |
| β/° | 96.412(2) | 84.757(4) | 96.329(2) | 96.357(2) | 94.032(3) |
| γ/° | 90 | 89.326(4) | 90 | 90 | 90 |
| V/Å$^3$ | 1096.85(8) | 963.57(16) | 1097.39(12) | 1097.83(9) | 2885.4(3) |
| Z | 2 | 1 | 2 | 2 | 2 |

**Table S10.** Sing crystal data of H-3, G-3A, H/G-3A

| Compound | H-3 | G-3A | H/G-3A | G-3B |
|---|---|---|---|---|
| CCDC no. | 2177005 | 2213152 | 2181832 | 2080763 |
| Formula | $C_{18}\ H_{10}\ F_4\ S_2$ | $C_{42}\ H_{30}\ S_6$ | $C_{18}\ H_{10}\ F_4\ S_2$ | $C_{30}\ H_{20}\ F_2\ S_4$ |
| Formular weight | 366.40 | 727.02 | 366.40 | 546.70 |
| Temperature/K | 173(2) | 173(2) K | 173(2) | 173(2) |
| Crystal system | Monoclinic | Triclinic | Monoclinic | Triclinic |
| Space group | $P2_1/n$ | $P\bar{1}$ | $P2_1/n$ | $P\bar{1}$ |
| a/Å | 15.5305(8) | 9.6077(2) | 15.5542(7) | 9.6203(3) |
| b/Å | 5.6743(3) | 10.2863(2) | 5.6674(2) | 14.1325(5) |
| c/Å | 18.5471(9) | 10.6653(2) | 18.5760(8) | 14.9666(5) |
| $\alpha/^o$ | 90 | 68.4380(10) | 90 | 76.2930(10) |
| $\beta/^o$ | 106.918(2) | 76.8880(10) | 106.9500(10) | 88.135(2) |
| $\gamma/^o$ | 90 | 65.4770(10) | 90 | 88.840(2) |
| $V/Å^3$ | 1563.72(14) | 888.40(3) | 1566.37(11) | 1975.68(11) |
| Z | 4 | 1 | 4 | 3 |

## Reference


1 T. Weng, G. Baryshnikov, C. Deng, X. Li, B. Wu, H. Wu, H. Ågren, Q. Zou, T. Zeng and L. Zhu, *Small,* 2020, **16**, 1906475.
2 S. Shen, G. Baryshnikov, B. Yue, B. Wu, X. Li, M. Zhang, H. Ågren and L. Zhu, *J. Mater. Chem. C,* 2021, **9**, 11707-11714.
3 T. Yanai, D. P. Tew and N. C. Handy, *Chem. Phy. Lett.,* 2004, **393**, 51-57.
4 E. Van Lenthe and E. J. Baerends, *J. Comput. Chem.,* 2003, **24**, 1142-1156.
5 G. te Velde, F. M. Bickelhaupt, E. J. Baerends, C. Fonseca Guerra, S. J. A. van Gisbergen, J. G. Snijders and T. Ziegler, *J. Comput. Chem.,* 2001, **22**, 931-967.
6 S. J. A. van Gisbergen, J. G. Snijders and E. J. Baerends, *Comput. Phys. Commun.,* 1999, **118**, 119-138.
7 E. van Lenthe, A. Ehlers and E.-J. Baerends, *J. Chem. Phys.,* 1999, **110**, 8943-8953.
8 E. van Lenthe, J. G. Snijders and E. J. Baerends, *J. Chem. Phys.,* 1996, **105**, 6505-6516.
9 F. Wang and T. Ziegler, *J. Chem. Phys.,* 2005, **123**, 154102.
10 V. G. Plotnikov, *Int. J. Quantum. Chem.,* 1979, **16**, 527-541.
11 G. V. Baryshnikov, R. R. Valiev, N. N. Karaush, V. A. Minaeva, A. N. Sinelnikov, S. K. Pedersen, M. Pittelkow, B. F. Minaev and H. Ågren, *Phys. Chem. Chem. Phys.,* 2016, **18**, 28040-28051.
12 R. R. Valiev, V. N. Cherepanov, V. Y. Artyukhov and D. Sundholm, *Phys. Chem. Chem. Phys.,* 2012, **14**, 11508-11517.
13 X. Jia, C. Shao, X. Bai, Q. Zhou, B. Wu, L. Wang, B. Yue, H. Zhu and L. Zhu, *Proc. Natl. Acad. Sci.,* 2019, **116**, 4816.
14 Z. Liu, K. Ouyang, and N. Yang, *Org. Biomol. Chem.,* 2018, **16**, 988.
15 G. Bergamini, A. Fermi, C. Botta, U. Giovanella, S. Di Motta, F. Negri, R. Peresutti, M. Gingras and P. Ceroni, *J. Mater. Chem. C*, 2013, **1**, 2717-2724.
16 T. Weng, Q. Zou, M. Zhang, B. Wu, G. Baryshnikov , S. Shen, X. Chen, H. Ågren, X. Jia and L. Zhu, *J. Phys. Chem. Lett.,* 2021, **12**, 6182-6189.
17 H. Wu, W. Chi, G. Baryshnikov, B. Wu, Y. Gong, D. Zheng, X. Li, Y. Zhao, X. Liu, H. Ågren and L. Zhu, *Angew. Chem. Int. Ed.*, 2019, **58**, 4328-4333.
18 N. Notsuka, R. Kabe, K. Goushi and C. Adachi, *Adv. Funct. Mater.,* 2017, **27**, 1703902.
19 S. Xu, W. Wang, H. Li, J. Zhang, R. Chen, S. Wang, C. Zheng, G. Xing, C. Song and W. Huang, *Nat. Commun.,* 2020, **11**, 4802.
20 Y. Lei, J. Yang, W. Dai, Y. Lan, J. Yang, X. Zheng, J. Shi, B. Tong, Z. Cai and Y. Dong, *Chem. Sci.,* 2021, **12**, 6518-6525.
21 Z. Xie, X. Zhang, H. Wang, C. Huang, H. Sun, M. Dong, L. Ji, Z. An and W. Huang, *Nat. Commun.*, 2021, **12**, 3522.
22 W. Zhao, T. S. Cheung, N, Jiang, W. Huang, J. W. Y. Lam, X. Zhang, Z. He and B. Z. Tang, *Nat. Commun.*, 2019, **10**, 1595.
23 J. Jin, H. Jiang, Q. Yang, L. Tang, Y. Tao, Y. Li, R. Chen, C. Zheng, Q. Fan, K. Y. Zhang, Q. Zhao and W. Huang, *Nat. Commun.,* 2020, **11**, 842.
